# Organizational culture in cardiovascular care in Chinese hospitals: a descriptive cross-sectional study

**DOI:** 10.1186/s12913-015-1211-7

**Published:** 2015-12-21

**Authors:** Emily S. Yin, Nicholas S. Downing, Xi Li, Sara J. Singer, Leslie A. Curry, Jing Li, Harlan M. Krumholz, Lixin Jiang

**Affiliations:** Department of Internal Medicine, Yale University School of Medicine, New Haven, CT USA; Section of Cardiovascular Medicine, Department of Internal Medicine, Yale University School of Medicine, New Haven, CT USA; National Clinical Research Center of Cardiovascular Diseases, State Key Laboratory of Cardiovascular Disease, Fuwai Hospital, National Center for Cardiovascular Diseases, Chinese Academy of Medical Sciences and Peking Union Medical College, Beijing, China; Department of Health Policy and Management, Harvard T.H. Chan School of Public Health, Boston, MA USA; Department of Medicine, Harvard Medical School, and Institute for Health Policy, Massachusetts General Hospital, Boston, MA USA; Robert Wood Johnson Foundation Clinical Scholars Program, Department of Internal Medicine, Yale University School of Medicine, New Haven, CT USA; Department of Health Policy and Management, Yale School of Public Health, New Haven, CT USA; Center for Outcomes Research and Evaluation, Yale-New Haven Hospital, New Haven, CT USA

**Keywords:** Survey, Hospitals, Organizational learning, Healthcare workers, Positive response

## Abstract

**Background:**

Organizational learning, the process by which a group changes its behavior in response to newly acquired knowledge, is critical to outstanding organizational performance. In hospitals, strong organizational learning culture is linked with improved health outcomes for patients. This study characterizes the organizational learning culture of hospitals in China from the perspective of a cardiology service.

**Methods:**

Using a modified Abbreviated Learning Organization Survey (27 questions), we characterized organizational learning culture in a nationally representative sample of 162 Chinese hospitals, selecting 2 individuals involved with cardiovascular care at each hospital. Responses were analyzed at the hospital level by calculating the average of the two responses to each question. Responses were categorized as positive if they were 5+ on a 7-point scale or 4+ on a 5-point scale. Univariate and multiple regression analyses were used to assess the relationship between selected hospital characteristics and perceptions of organizational learning culture.

**Results:**

Of the 324 participants invited to take the survey, 316 responded (98 % response rate). Perceptions of organizational learning culture varied among items, among domains, and both among and within hospitals. Overall, the median proportion of positive responses was 82 % (interquartile range = 59 % to 93 %). “Training,” “Performance Monitoring,” and “Leadership that Reinforces Learning” were characterized as the most favorable domains, while “Time for Reflection” was the least favorable. Multiple regression analyses showed that region was the only factor significantly correlated with overall positive response rate.

**Conclusions:**

This nationally representative survey demonstrated variation in hospital organizational learning culture among hospitals in China. The variation was not substantially explained by hospital characteristics. Organizational learning culture domains with lower positive response rates reveal important areas for improvement.

**Electronic supplementary material:**

The online version of this article (doi:10.1186/s12913-015-1211-7) contains supplementary material, which is available to authorized users.

## Background

Organizational learning is defined as the process by which a group changes its behavior in response to newly acquired knowledge [1]. Organizational learning involves the institutionalization of knowledge and creation of organizational memory, such that the knowledge base of a group is greater than the sum of its individual parts [2]. Organizational learning balances exploration (feed-forward, knowledge-seeking behavior) with exploitation (feedback, knowledge-applying behavior) and is critical to outstanding organizational performance [3]. Strong organizational learning practices enable high functioning by fostering a safe learning environment. Through actions such as providing time for reflection and encouraging information sharing, organizations may engage in increased problem solving, thereby producing useful knowledge that can be used to formulate effective quality improvement initiatives [4–8].

Employing organizational learning to improve performance is widely applicable across industries: from automobile manufacturers to IT corporations, organizations are increasingly emphasizing learning culture as a means of enhancing productivity, product quality, and cost-effectiveness [9–11]. Learning can also occur between industries, with teams adapting new methodologies from outside of their own fields [12]. Several instruments have been developed to assess perceptions of organizational learning. The use of these organizational learning assessment tools in quality improvement initiatives rests on three assumptions: (1) within hospital teams, there exist discernable cultures that affect quality and performance; (2) these cultures are malleable; and (3) specific cultural features are related to performance, and thus it is possible to strategically alter culture to effect an improvement in performance [13]. Determining the underlying causes of productivity differences among organizations can provide less efficient organizations with the knowledge necessary to achieve outstanding performance [14].

Organizational learning is particularly important in highly competitive industries and industries with rapidly changing knowledge bases [15]. In hospitals, which fall into the latter category, there is significant room for improvement in performance [16, 17]. Organizational learning is critical in health care settings, as patient outcomes depend on provider performance [18]. A number of organizational learning practices and aspects of learning culture have been associated with improved patient care; among them include team motivation to change, encouraging open discussion, and creating learning-oriented goals rather than performance-oriented goals [19–22]. Organizational learning cultures that support learning are also linked with lower rates of adverse events and readmission [23, 24].

For health care organizations in low- and middle- income countries, improving organizational learning culture is a particularly essential goal. In health care systems that are limited in their ability to use costly drugs and technologies, achieving superlative performance may only be possible through a hospital culture that maximizes the impact of available resources [25, 26]. Learning strategies that allow hospital personnel to deliver more effective and efficient care may be especially important in resource-limited systems [27, 28]. In today’s era of global communication and knowledge exchange, these goals have never been more achievable.

China is a country with particular health care challenges. Recent reforms have driven a massive increase in demand for health care, necessitating more efficient delivery of care [29, 30]. Achieving excellence in care using existing resources may have implications for millions of patients in a country that comprises nearly one-fifth of the world’s population [31, 32]. This issue has particular salience for cardiovascular care, in which the delivery of services can be costly. Determining specific aspects of learning culture that can be improved upon is necessary to inform the development of initiatives to improve care [33]. Therefore, a national assessment of organizational learning culture in cardiovascular services is an important step in developing quality improvement initiatives. Although some studies have analyzed similar aspects of patient safety culture or organizational culture in Chinese hospitals, we know of no nationally representative study characterizing organizational learning culture in China and none specifically focusing on cardiovascular care [24, 34–37].

Accordingly, we characterized the organizational learning environment of hospitals in China from the perspective of individuals providing cardiovascular care. We focused the survey on care for patients with acute myocardial infarction (AMI), a leading cause of death in China and a disease that has an increasingly large burden in developing nations [38–40]. Here, we report the first use of the previously validated Short Form Learning Organization Survey in Mandarin Chinese [41]. We administered the survey to hospital staff to assess perceptions of hospitals’ organizational learning cultures across the nationally representative network of hospitals established by the China Patient-centered Evaluative Assessment of Cardiac Events (China PEACE) studies [42]. This study lays the groundwork for future analysis of these organizational features with respect to patient outcomes.

## Methods

### Sampling design

The hospital sampling design of the China PEACE-Retrospective Acute Myocardial Infarction Study, a study assessing trends in AMI care in China over the past decade, has been described previously [42]. Briefly, a nationally representative sample of hospitals in China was developed by random sampling in 5 strata: Eastern-rural, Central-rural, Western-rural, Eastern-urban, and Central/Western-urban regions. These strata were used because economic development and health care resources differ between urban and rural areas as well as among the 3 official economic-geographic regions (Eastern, Central, and Western) of Mainland China. Central and Western urban regions were grouped together given their similar per capita income and health services capacity [43].

In the three rural strata, the sampling framework consisted of the central hospital in each of the predefined rural regions (2010 central hospitals in 2010 rural regions). In the two urban strata, the sampling framework consisted of the highest level hospitals in each of the predefined urban regions (833 hospitals in 287 urban regions). Hospital level is defined by the Chinese government based on clinical resource capacity: secondary hospitals have >100 inpatient beds, acute medical care, and preventative care services for populations of >100,000, whereas tertiary hospitals are larger referral centers in provincial capitals and major cities. A representative sample of non-military hospitals from 2011 was randomly selected to reflect current practices, with 35 hospitals in each stratum. Prison hospitals, specialized hospitals without a cardiovascular disease division, and traditional medicine hospitals were excluded (Additional file 1: Figure S1). Seven hospitals were excluded for a lack of AMI cases, and 6 of the remaining hospitals declined to participate. In total, 162 hospitals participated in the China PEACE-Retrospective AMI Study.

### Selection of participants

We identified two individuals from each hospital for participation in the survey: the principal investigator (PI) and the coordinator of the China PEACE-Retrospective AMI Study (“study coordinator”). To minimize social desirability bias, we did not inform either participant of the other’s involvement [44]. The definitions of the roles were established during the planning phase of the China PEACE-Retrospective AMI Study; typically, the director of the Cardiology Department or Internal Medicine Department at each hospital served as the principal investigator, and the China PEACE study coordinator was most often a physician selected by the principal investigator. We invited principal investigators to participate in the China PEACE-Retrospective AMI Study via phone or email. When direct contact information could not be obtained by our staff, the lead regional investigator for the China PEACE-Retrospective AMI Study connected our staff to the individual who was appropriate to serve as principal investigator. Subsequently, the principal investigator selected a member of his or her team to serve as the hospital’s coordinator for the China PEACE-Retrospective AMI Study. We therefore did not specifically choose participants for the survey study, but rather invited previously selected individuals to participate. Additionally, we believe that their coordinating roles may afford these individuals a valuable perspective on the organizational learning cultures of their hospitals.

### Survey design

We organized the survey into 4 sections: personal information of the respondent (part A); general information about the hospital and the department in charge of AMI care (part B); information about hospital practices relating to the diagnosis and treatment of cardiovascular heart disease (part C); and organizational learning characteristics and quality improvement for AMI care (part D) (Additional file 2: Survey).

Organizational learning culture was measured in Part D, which drew questions from the Short-Form Learning Organization Survey (LOS-27, an abbreviated version of the original Garvin et al. Learning Organization Survey) and the Survival after AMI (SAMI) study. The questions derived from the LOS-27 represent the primary focus of this paper [4, 41]. The LOS-27 consists of 27 questions, grouped into 7 domains of organizational learning characteristics (Additional file 1: Table S1) [41].

The survey also included questions pertaining to quality improvement in AMI care adapted from the SAMI instrument, which was developed through qualitative research with hospital staff to determine organizational characteristics that differentiated high-performing from low-performing hospitals on the basis of 30-day risk-standardized mortality rates for AMI [45, 46]. The questions from the SAMI survey are grouped into 6 domains, some of which overlap with the original Garvin survey: problem solving and learning; organizational values and goals; senior management involvement; communication and coordination among groups; hospital protocols and practices to improve AMI care; and broad staff presence and expertise in AMI care [45].

The survey was written in English and translated into Mandarin Chinese. To ensure accuracy, a double translation [47] was conducted in which the survey was translated into Chinese and then back into English independently by two bilingual Chinese medical researchers. Modifications were made to the Chinese translation accordingly. Participants were informed at the start of the survey that their responses would be used to study institutional characteristics and medical care patterns. The central ethics committee at the China National Center for Cardiovascular Disease approved the PEACE-Retrospective AMI Study. All collaborating hospitals accepted the central ethics approval except for the following five hospitals, which obtained approval from their respective internal ethics committees: Affiliated Zhongshan Hospital of Dalian University, First Hospital of Tsinghua University, First People’s Hospital of Guangyuan, Jilin Province People’s Hospital, and Tianjin Medical University General Hospital.

### Survey pilot and refinement

The survey was piloted using a convenience sample of 6 hospitals with percutaneous coronary intervention capability. The principal investigators were invited to participate in the pilot, and one study coordinator also volunteered to participate. The responses of the six principal investigators (3 via in-person interviews and 3 via self-administered paper-based survey) and one study coordinator (via self-administered paper-based survey) were collected. The cognitive interviewing methodology [48], in which individual interviews are conducted with each pilot participant, was used to assess understanding of the pilot survey. For paper-based pilot surveys, cognitive interviewing consisted of retrospective (post-survey) probes; for in-person interviews, concurrent (during survey) probes allowed participants to provide survey feedback in real-time. Based on the experience from the pilot, minor revisions were made to clarify the meaning of certain questions (i.e., altering “很容易 (can easily)” to “可以 (are able to)”), and the sequence of questions was modified to improve logic and flow. No questions were removed or added. All data from the pilot testing were included in the final data set.

### Survey data collection and processing

The survey was available in two forms: web-based e-survey, in which each participant was able to log in with a unique password to a website where the survey was hosted, and PDF-based survey, in which subjects digitally marked their answers in PDF files and returned the files via email. We applied two methods to ensure the quality of the responses [49]. We checked the response data for completeness, either by automatic verification (web-based) or by manual check by our staff (PDF-based), and on the basis of logic. For the web-based e-survey submissions, we recorded total time spent on the survey and used automatic logic check and verification while subjects were responding to the survey. For the PDF-based survey submissions, we conduced a manual logic check, focusing on whether subjects correctly skipped inapplicable questions as indicated by the instructions in other parts of the survey. In cases of missing or illogical (e.g., questions incorrectly skipped or completed) data for PDF-based surveys, we contacted respondents by email and/or phone, informed them of which questions needed to be resolved, and asked them to resubmit the survey with the necessary changes.

### Data analysis

We scored all survey items on either a 7-point scale of statement accuracy (1-*Highly inaccurate* to 7-*Highly accurate*) or a 5-point scale of frequency (1-*Never* to 5-*Always*). Negatively phrased question scores were reversed to ensure that the highest score always corresponded with the strongest positive response. At hospitals with two complete response sets, we analyzed responses at the hospital level by averaging the two responses to each question in order to calculate a representative value for the hospital. Subsequently, we determined whether this average value, or raw response in the case of the hospitals from which only 1 completed survey was received, represented a positive response (e.g., a favorable organizational learning culture): for those questions scored on a 7-point scale, we categorized the highest three scores (i.e., 5 to 7) as positive responses, while for 5-point scale items, we categorized the highest two scores (i.e., 4 and 5) as positive responses. Non-integer averaged scores were rounded up.

We defined four summary statistics to describe the results of the survey in aggregate. Hospital positive response rate (hospital PRR) was calculated at each hospital as the proportion of questions that had a positive response. Survey question positive response rate (survey question PRR) was calculated for each survey item as the proportion of hospitals with a positive response as defined above. Domain positive response rate (domain PRR) was calculated at each hospital as the number of questions within a given domain for which the hospital had an overall positive response.

Using descriptive statistics, we characterized the hospitals and individual participants involved in the study, as well as the hospital organizational learning culture. We then used Wilcoxon tests to assess overall and domain-specific differences among hospitals based on hospital characteristics. We conducted univariate and multiple regression analyses to assess the relationships between hospital characteristics (hospital level, region, and urban/rural location) and hospital-level PRRs, including overall PRR, the domain with the lowest PRR, and the question with the lowest PRR. We used the PRRs for the survey domain and question with the lowest average PRRs in order to assess variation in organizational learning areas with the greatest room for improvement. Regression analyses were performed using Stata 12.0 (StataCorp LP). All other analyses were performed using Microsoft Excel for Mac 2011 and JMP version 10.0.0 (SAS Institute Inc).

## Results

### Characteristics of the sample

Of the 162 participating hospitals in the China PEACE-Retrospective AMI Study, more than half (61.1 %) were located in rural areas (Table 1A). The regional distribution of these hospitals across the Eastern, Central, and Western regions of China were 39.5 %, 29.6 %, and 30.9 %, respectively. Tertiary-level hospitals comprised 40.1 % of the sample, while 59.9 % were secondary level or below.

**Table 1:** Characteristics of the sample

Panel A: Characteristics of participating hospitals			Panel B: Characteristics of respondents		
	#	%		#	%
ALL HOSPITALS	162	100.0	ALL RESPONDENTS	316	100.0 %
RURAL/URBAN LOCATION			GENDER		
Urban	63	38.9 %	Male	237	75.0 %
Rural	99	61.1 %	Female	79	25.0 %
GEOGRAPHIC REGION			EDUCATION		
Eastern	64	39.5 %	College (junior college)	222	70.3 %
Central	48	29.6 %	Postgraduate	94	29.7 %
Western	50	30.9 %	CLINICAL JOB TITLE^†^		
HOSPITAL LEVEL			Consultant (i.e., directors)	182	57.6 %
Secondary or below	97	59.9 %	Attendant	85	26.9 %
Tertiary	65	40.1 %	Resident	41	13.0 %
TEACHING STATUS			Nurse	4	1.3 %
Teaching	68	42.0 %	Other (non-clinical)	4	1.3 %
Non-teaching	53	32.7 %	SENIOR ADMINISTRATIVE POSITION IN HOSPITAL		
No response	41	25.3 %	Yes	198	62.7 %
			No	118	37.3 %
			NUMBER OF YEARS WORKING IN DEPARTMENT^‡^		
			x ≤ 5	67	21.2 %
			5 < x ≤10	57	18.0 %
			10 < x ≤ 20	106	33.5 %
			>20	85	26.9 %

^†^Percentages do not add to one hundred due to rounding

^‡^Percentages do not add to one hundred because one respondent did not answer this question

Of the respondents, three-quarters (75.0 %) were male (Table 1B). The majority (97.5 %) were physicians, of whom 57.6 % were consultant physicians (highest level) and 39.9 % were physicians-in-training (resident level or above); 2.5 % of respondents were nurses or non-clinical staff. Of the 162 hospitals, 154 (95.1 %) had 2 complete survey responses while the remaining 8 had 1 complete survey response.

### Survey completion

**Fig. 1:**
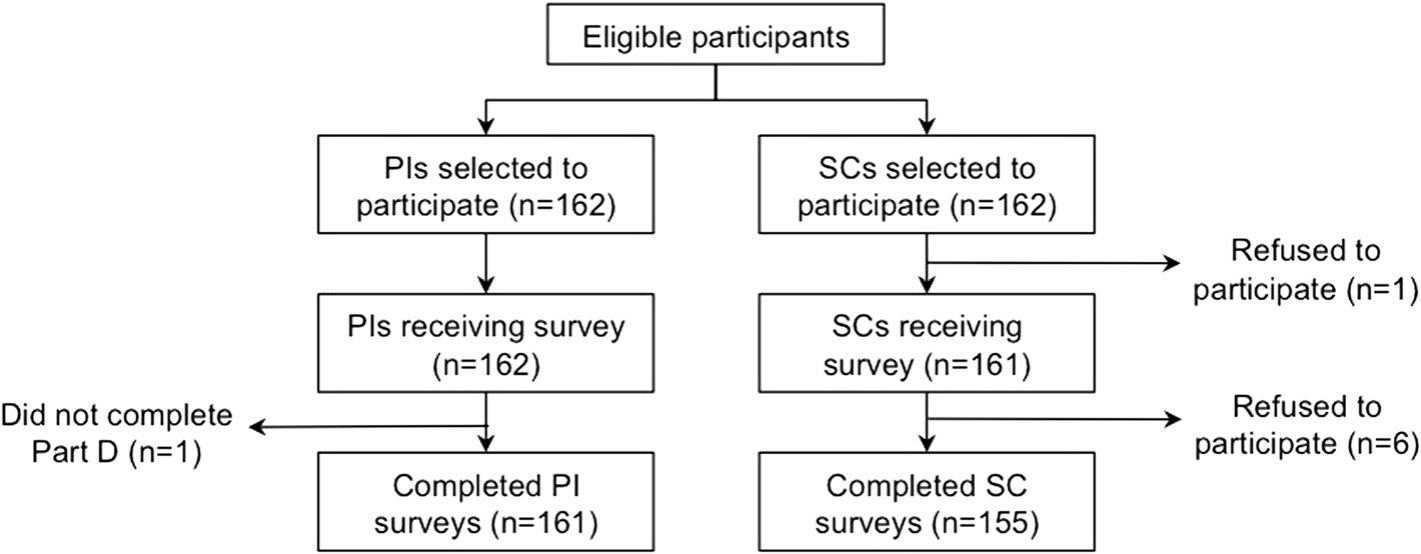
Study design showing participant selection. Of the 324 eligible participants at each hospital (i.e., the pre-selected principal investigators (PIs) and study coordinators (SCs) of the China PEACE project), 162 PIs and 155 SCs agreed to participate. Of these, 161 PIs and all 155 SCs completed all organizational learning questions derived from the Learning Organization Survey (LOS)-27

Of the 324 individuals invited to participate in the survey, 317 submitted responses (97.8 % response rate; Fig. 1). Of these, 90 submissions (28.4 %) required follow-up contact to resolve missing or illogical responses (Additional file 1: Appendix Supplement). After the exclusion of one respondent who failed to complete the organizational learning component (Part D) of the survey, there were 316 complete responses for the 27 questions that comprise the LOS-27 in Part D of the survey (97.5 % response rate).

### Individual questions

The median survey question PRR was 74.1 % (IQR: 62.3-78.4). The survey item with the highest proportion of positive responses (93.8 %) was, *This workgroup consistently collects information on technological trends*, within the “Performance Monitoring” domain. The survey item with the lowest proportion of positive responses (survey question PRR = 43.2 %) was, *This workgroup has forums for meeting with and learning from: Customers/clients* within the “Knowledge Acquisition” domain (Additional file 1: Figure S2).

### Variation in overall organizational learning culture among hospitals

The median hospital PRR was 81.5 % (IQR: 59.3-92.6; Additional file 1: Table S2). There were 30 hospitals (18.5 %) with a hospital PRR of less than 50 %, and 72 hospitals (44.4 %) with a hospital PRR of less than 75 % (Additional file 1: Table S3). There were 6.8 % of hospitals that had overall hospital PRRs of 100 %, meaning that for every item on the survey, the averaged hospital response was positive. Hospital PRR also varied with hospital-level characteristics (Additional file 1: Table S2 and Table S4). Of the hospital-level factors assessed, region was most strongly associated with organizational learning culture (*P* = 0.001), with less positive organizational learning culture in Western China (Additional file 1: Table S2). Urban/rural location and government-defined hospital level were also associated with organizational learning culture with borderline significance (*P* = 0.017 and 0.011, respectively).

### Variation in organizational learning culture by domain

**Fig 2:**
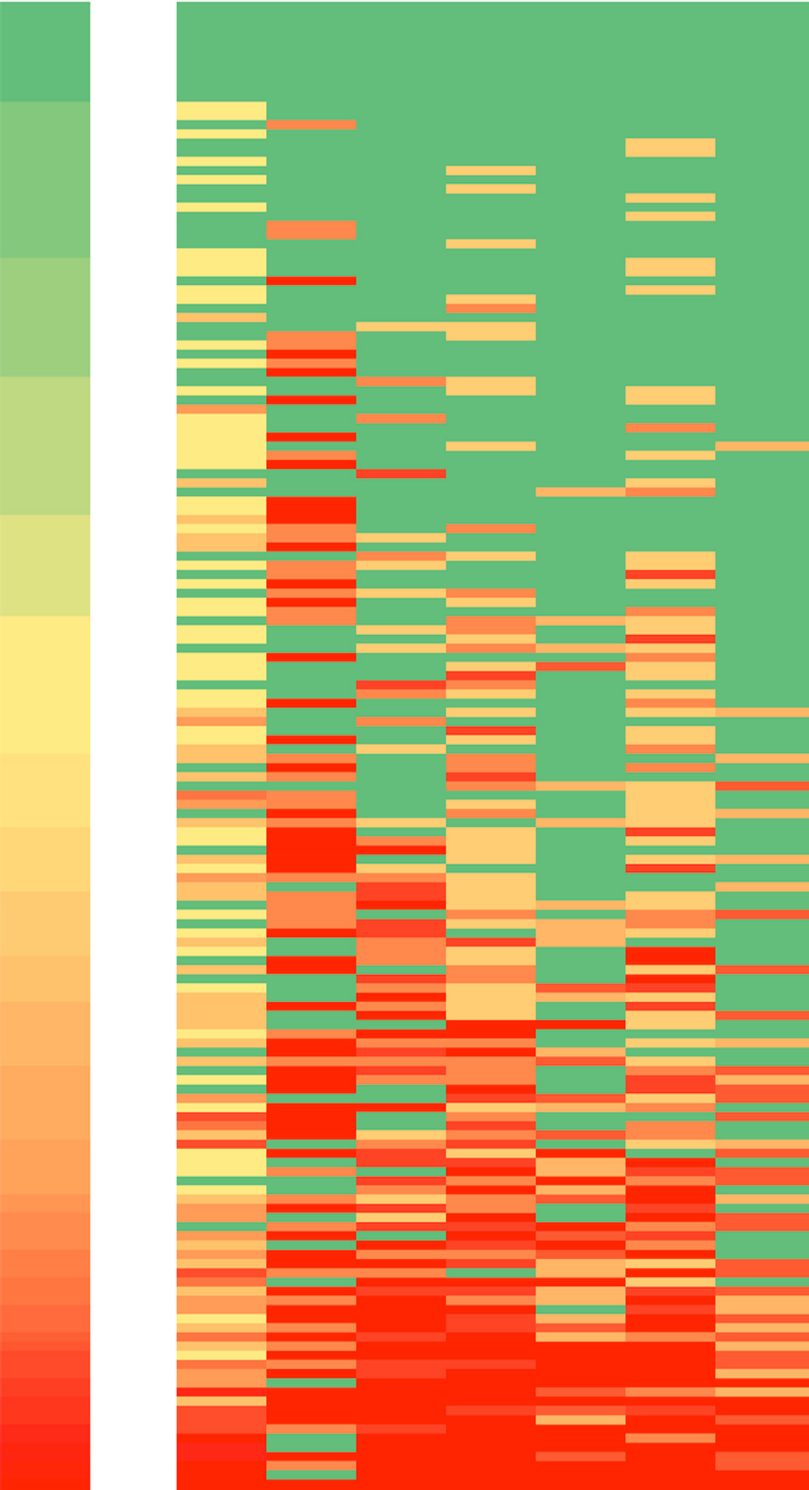
Heat map of positive response rates. Heat map of domain-specific positive response rates (PRR) by hospital. Cell colors range on a scale from green (PRR = 100 %) to red (PRR = 0 %), where yellow represents the median. Each row represents 1 hospital. The left-most column represents the PRR of each hospital. Each of the following 7 columns represents an LOS-27 domain; from left to right: supportive learning environment, time for reflection, leadership that reinforces learning, experimentation, training, knowledge acquisition, and performance monitoring. Rows were sorted by overall PRR, with the hospital with the highest overall PRR displayed at the top and the hospital with the lowest overall PRR at the bottom

Domain PRR differed across the seven domains of the LOS-27 (*P* < 0.0001, Fig. 2). The median domain PRR was 100 % in 3 domains: “Training” (IQR: 66.7-100.0), “Performance Monitoring” (IQR: 66.7-100.0), and “Leadership that Reinforces Learning” (IQR: 25.0-100). The domain with the lowest domain PRR was “Time for Reflection” (median PRR 50 %; IQR: 0.0-100); the questions in this domain describe whether an organization dedicates time towards reflecting on past work and future quality improvement. Within this domain, 48.8 % of hospitals agreed with the statement that, “There is simply no time for reflection.”

### Univariate and multiple regression analyses

Table 2 shows the results of the univariate regression model. Our model indicates that eastern region (0.162, 95 % CI [0.072, 0.251], *P* < 0.001) was associated with higher overall PRR, whereas rural location (−0.085, 95 % CI [−0.162, −0.007], *P* = 0.033), and secondary hospital level (−0.079, 95 % CI −0.157, −0.002], *P* = 0.045) were associated with lower overall PRR. Only eastern region (0.166, 95 % CI [0.005, 0.328], *P* = 0.044) correlated with the “Time for Reflection” domain PRR. For the question with the lowest PRR, *This workgroup has forums for meeting with and learning from: Customers/clients,* none of the three hospital characteristics assessed were significantly contributory.

**Table 2:** Univariate analysis of hospital characteristics and PRRs

	Overall PRR		Time for Reflection		Customers/Clients*	
Covariates	Point estimate (95 % CI)	*P* value	Point estimate (95 % CI)	*P* value	Point estimate (95 % CI)	*P* value
Level						
Tertiary	Reference	--	Reference	--	Reference	--
Secondary or below	−0.079 (−0.157, −0.002)	0.045	−0.105 (−0.243, 0.033)	0.134	−0.063 (−0.520, 0.395)	0.787
Region						
Western	Reference	--	Reference	--	Reference	--
Central	0.083 (−0.013, 0.178)	0.090	0.041 (−0.132, 0.214)	0.638	−0.004 (−0.579, 0.571)	0.990
Eastern	0.162 (0.072, 0.251)	<0.001	0.166 (0.005, 0.328)	0.044	0.340 (−0.197, 0.877)	0.213
Location						
Urban	Reference	--	Reference	--	Reference	--
Rural	−0.085 (−0.162, −0.007)	0.033	−0.136 (−0.274, 0.002)	0.054	−0.118 (−0.577, 0.342)	0.614

*“Customers/Clients” corresponds to the survey question “This workgroup has forums for meeting with and learning from: Customers/clients”

Our multiple regression model (Table 3) showed that only eastern region (0.152, 95 % CI 0.062, 0.242], *P* = 0.001) was significantly correlated with overall PRR. None of the three hospital characteristics assessed were significantly correlated with the PRR for the “Time for Reflection” domain or the PRR for *This workgroup has forums for meeting with and learning from: Customers/clients*. The three variables only explained a small proportion of variation in the PRR (R-square <0.1 across different models).

**Table 3:** Multiple analysis of hospital characteristics and PRRs

	Overall PRR		Time for Reflection		Customers/Clients*	
Covariates	Point estimate (95 % CI)	*P* value	Point estimate (95 % CI)	*P* value	Point estimate (95 % CI)	*P* value
Level						
Tertiary	Reference	--	Reference	--	Reference	--
Secondary or below	−0.003 (−0.146, 0.140)	0.966	0.067 (−0.193, 0.327)	0.614	0.231 (−0.640, 1.102)	0.601
Region						
Western	Reference	--	Reference	--	Reference	--
Central	0.089 (−0.007, 0.184)	0.068	0.049 (−0.124, 0.222)	0.576	−0.007 (−0.587, 0.573)	0.981
Eastern	0.152 (0.062, 0.242)	0.001	0.155 (−0.009, 0.318)	0.063	0.348 (−0.199, 0.896)	0.210
Location						
Urban	Reference	--	Reference	--	Reference	--
Rural	−0.067 (−0.209, 0.075)	0.352	−0.171 (−0.429, 0.087)	0.192	−0.249 (−1.112, 0.615)	0.570

*“Customers/Clients” corresponds to the survey question “This workgroup has forums for meeting with and learning from: Customers/clients”

## Discussion

In the China PEACE Hospital Survey Study on Organizational Learning, we characterized the organizational learning cultures of a nationally representative sample of hospitals in China. While hospitals had an overall positive perception of organizational learning culture, there was significant variability among hospitals. Eastern region was also significantly correlated with more positive perceptions of organizational learning.

In this study, low domain PRRs indicated areas of organizational learning culture in which there is significant room for improvement. Nearly half of all hospitals agreed that “there is simply no time for reflection” in their workplace. This finding supports concerns that limited time for reflection, a widespread issue in health care environments, may hinder organizational learning [50]. A lack of time for reflection may impede performance by limiting engagement in quality improvement activities, such as team discussions and problem solving.

Conversely, we observed that more than half of hospitals had the highest possible PRRs in the organizational learning culture domains of “Training,” “Performance Monitoring,” and “Leadership that Reinforces Learning.” These findings, respectively, suggest that Chinese hospital teams do well at providing adequate training for their members, using outside information to assess performance, and reinforcing strong learning habits through leadership. The “Performance Monitoring” domain also contained the question with the highest PRR (“This workgroup consistently collects information on technological trends,” PRR = 93.8 %). This phenomenon is supported by findings that Chinese health care providers highly value technological skill and its influence on patient care [51]. However, we were unable to directly compare the organizational learning culture of China with that of the US or other countries, as the LOS-27 has thus far only been used in a validation study.

This study also identified specific areas in which hospitals can improve organizational learning culture. We found that patients are often not formally consulted for quality improvement, as evidenced by the question with the lowest PRR, “This workgroup has forums for meeting with and learning from: Customers/clients.” This supports descriptions of a discontinuity between hospital managers’ belief in the importance of patient experience and their failure to provide structured plans for its improvement [52]. Resolving this discrepancy by soliciting and discussing patient feedback may help improve the patient experience [53]. In contrast, many hospitals readily had forums for meeting with experts outside the organization and experts from other departments/teams/divisions (PRR = 77.2 % and 73.5 %, respectively). Although the absence of formal “forums” for meeting with patients does not necessarily indicate that staff do not consult patients for feedback, it suggests that this may be an area deserving of greater attention.

In addition, our study indicates that hospital characteristics only partially account for the variation in PRR. While region, urban/rural location, and hospital level may affect organizational learning culture, other contributing factors may include the medical capabilities of the hospital, staff-to-patient ratios, and attitudes of hospital management toward organizational learning.

Overall, our study provides an analysis of organizational learning culture in a representative sample of Chinese hospitals and shows significant variation in organizational learning culture among hospitals, indicating room for improvement. Evidence connecting organizational learning with high performance across industries suggests that health care organizations and their patients stand to benefit from strong organizational learning cultures – a relationship that has been observed in studies of US hospitals [45, 54, 55]. Our analysis highlights the strengths and weaknesses of hospital organizational learning cultures in China; this assessment may enable the development of specific strategies for improvement, such as setting aside dedicated time for reflection and feedback, creating structured methods or forums for collaboration, and training managers to support organizational learning habits. However, the relationship between organizational learning culture and clinical outcomes has yet to be studied in China.

Although our survey draws from thoroughly developed instruments in the literature, the study has some limitations. First, despite double translation by Chinese medical professionals and cognitive interviewing to minimize misinterpretation of the questions [48], the Chinese translations of the LOS-27 questions have not been formally validated. Therefore, we cannot verify that the questions are valid representations of the organizational learning domains as was confirmed in English. Second, our results may be affected by social desirability bias, in which respondents misrepresent their opinions in order to provide what they believe is a desirable response. We aimed to have two participants from each hospital in order to minimize any single participant’s bias, and we contacted respondents individually to participate in the study for their privacy. Third, two individuals’ perceptions of organizational learning culture cannot accurately represent the views of all team members at a given hospital. However, use of a subset for surveying is a common approach in organizational culture [13]. In addition, our sample, which was composed largely of higher level clinicians (57.6 % consultant-level physicians vs. 1.3 % nurses), may have inflated the actual perception of organizational learning culture; senior managers have been shown to be more optimistic than frontline workers regarding safety climate [56]. To minimize these effects, our study focused primarily on relative differences (e.g., variation). Although we did not adjust results for role (principal investigator vs. staff coordinator), the adjustment likely would not have had a significant effect, given the high response rate and inclusion of two participants at the majority of hospitals. Lastly, even hospitals with 100 % PRR do not have perfect organizational learning cultures, as “somewhat agree,” “agree,” and “strongly agree” are considered equally here as positive responses. However, this further supports the notion that there is significant room for improvement of organizational learning habits despite the overall positive culture.

## Conclusions

Our study showed that respondents reported an overall positive organizational learning culture in hospitals in China. Among a representative group of hospitals in China, we found significant and substantial variation in organizational learning culture and an association between organizational learning culture and hospital characteristics such as region. This suggests that the geographical location of a hospital may affect the organizational context in which it functions. However, there may be other factors more predictive of variation in organizational learning cultures that we have not yet assessed. Our study also provides evidence that there are opportunities for organizational learning improvements in China, and identifies specific areas in which hospitals can improve their organizational learning cultures, such as providing staff with sufficient time for reflection. These findings will help lay the groundwork for future quality improvement initiatives aimed at enhancing the organizational learning culture in Chinese hospitals.

## Additional files

Additional file 1: Appendix. Supplementary figures and tables. (DOCX 738 kb) Additional file 2:
**Survey.** China PEACE hospital survey in both English and Chinese. (PDF 427 kb)

## Abbreviations

AMI: Acute myocardial infarction
China PEACE: China Patient-centered Evaluative Assessment of Cardiac Events
LOS: Learning Organization Survey
PRR: Positive response rate
SAMI: Survival after AMI
