# Supplementary material for: Organizational culture in cardiovascular care in Chinese hospitals: a descriptive cross-sectional study
Source: BMC Health Serv Res. 2015 Dec 21;15:569. doi: 10.1186/s12913-015-1211-7 (PMC4685633; doi:10.1186/s12913-015-1211-7)
Supplement: Additional file 1: Appendix. — Supplementary figures and tables. (DOCX 738 kb) [file 12913_2015_1211_MOESM1_ESM.docx]

# Additional file 1 – Appendix

## Appendix Supplement.

In the case of missing or illogical data, respondents were contacted by phone and/or email to resolve the issue. In 6 cases, missing data could not be resolved after repeated contact by telephone, due to respondent refusal. The chief at hospital 941 failed to complete the entirety of Part D of the survey. The other missing responses were isolated data points (D48 in 841’s staff, D70 in 841’s staff, D43_4 in 943’s chief, D44_3 in 833’s staff, and C11 in 833’s chief).

## Figure S1 - Hospital sampling strategy.

Flowchart showing the hospital sampling strategy of the China PEACE research network. The resulting 162 hospitals participated in this survey study.

AMI, acute myocardial infarction

**
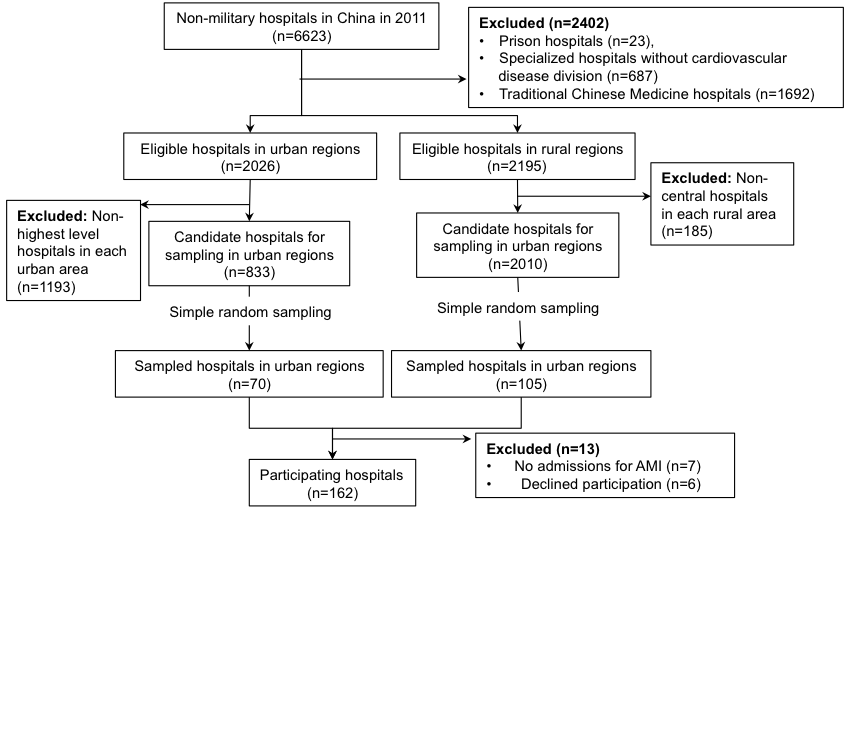
**Figure S2 - Breakdown of responses per question by domain.

Percentage of participants (y-axis) providing a given response (x-axis) for each survey question. Questions are grouped by organizational learning domain as defined by the LOS-27. The responses from all 317 survey respondents are represented, including those of the 1 respondent who failed to complete Part D of the survey.

LOS, Learning Organization Survey


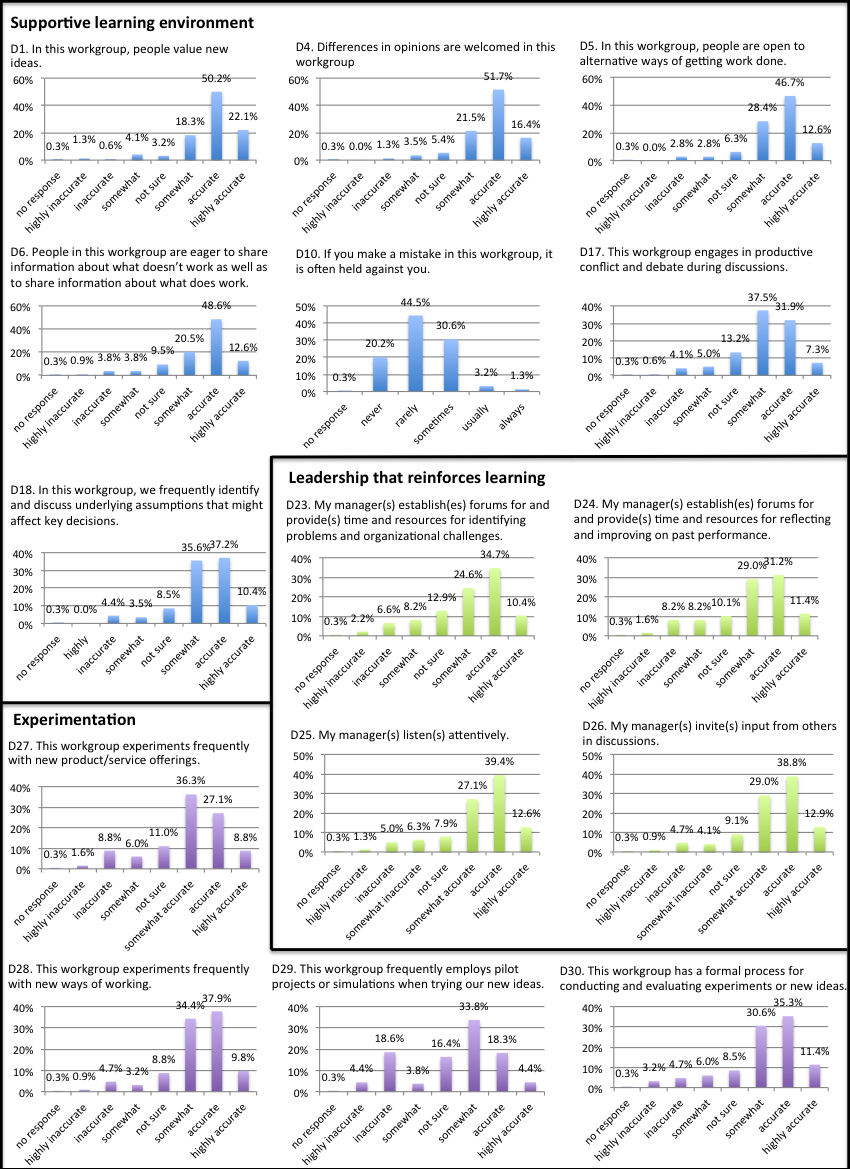


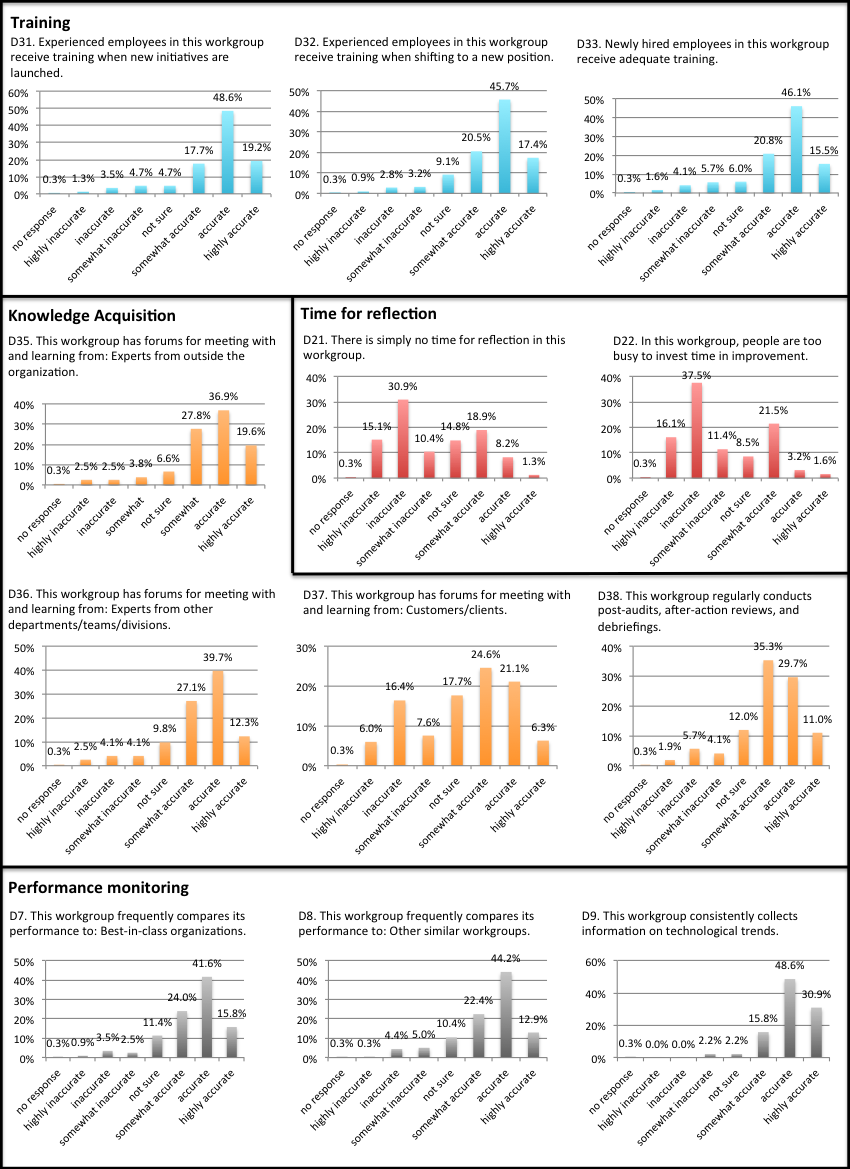


## Table S1 – Learning Organization Survey (LOS) domains.

The LOS-27 is composed of 7 domains of organizational learning: supportive learning environment; time for reflection; leadership that reinforces learning; experimentation; training; knowledge acquisition; and performance monitoring.[1] These domains were derived from three “building blocks” and nine sub-domains outlined by Garvin et al.[2] To reduce redundancy and enhance validity in a healthcare setting, questions were re-distributed into 7 domains using exploratory factor analysis. Question responses were scaled from 1-7 (Highly inaccurate, Inaccurate, Somewhat inaccurate, Neither accurate nor inaccurate, Somewhat accurate, Accurate, Highly accurate) or 1-5 (Never, Rarely, Sometimes, Usually, Always).

|  | **Characteristics** | **Question Example (scale)** |
| --- | --- | --- |
| **Supportive learning environment** | Members:   - Are comfortable with raising disagreements - Feel safe admitting to mistakes - Appreciate opposing ideas | This workgroup engages in productive conflict and debate during discussions. (1-7) |
| **Time for reflection** | Members:   - Have sufficient time to reflect - Have time to invest in improvement | There is simply no time for reflection in this workgroup. (1-7) |
| **Leadership that reinforces learning** | Leaders:   - Allow for input and discussion - Provide structured means for identifying problems | My manager(s) establish(es) forums for and provide(s) time and resources for reflecting and improving on past performance. (1-7) |
| **Experimentation** | The workgroup:   - Experiments with new ideas or methods | This workgroup frequently employs pilot projects or simulations when trying our new ideas. (1-7) |
| **Training** | - New employees receive adequate training - Experienced employees continue to receive training regularly or when new skills are necessary | Experienced employees in this workgroup receive training when shifting to a new position. (1-7) |
| **Knowledge acquisition** | The team has formal processes for:   - Sharing information - Acquiring information from experts and customers | This workgroup has forums for meeting with and learning from: Experts from outside the organization. (1-7) |
| **Performance monitoring** | The team has formal processes for:   - Gathering and interpreting outside information - Monitoring technological trends | This workgroup frequently compares its performance to: Best-in-class organizations. (1-7) |

## Table S2 - Overall PRR by domain and hospital level factors.

Indicates values of: Median (IQR) [Range]. P-values are shown for chi-squared tests between domain positive response rates (PRRs) by hospital-level factors. Domains are abbreviated as follows: SLE = Supportive learning environment; TFR = Time for reflection; LRL = Leadership that reinforces learning; E = Experimentation; T = Training; KA = Knowledge acquisition; PM = Performance monitoring.

|  | **n** | **All domains** | **SLE** | **TFR** | **LRL** | **E** | **T** | **KA** | **PM** |
| --- | --- | --- | --- | --- | --- | --- | --- | --- | --- |
| All hospitals | 162 | 81.5% (59.3%-92.6%) [9.3%-100%] | 85.7% (71.4%-100%)  [0%-100%] | 50.0% (0.0%-100%)  [0%-100%] | 100.0% (25.0%-100%)  [0%-100%] | 75.0% (25.0%-100%)  [0%-100%] | 100.0% (66.7%-100%)  [0%-100%] | 75.0% (50.0%-100%)  [0%-100%] | 100.0% (66.7%-100%)  [0%-100%] |
| **Urban/rural** | |  |  |  |  |  |  |  |  |
| Urban | 63 | 85.2%  (70.4%-92.6%) | 85.7%  (71.4%-100%) | 100.0%  (50.0%-100%) | 100.0%  (25.0%-100%) | 75.0%  (50.0%-100%) | 100.0%  (66.7%-100%) | 75.0%  (50.0%-100%) | 100.0%  (100%-100%) |
| Rural | 99 | 74.1%  (55.6%-88.9%) | 85.7%  (71.4%-100%) | 50.0%  (0.0%-100%) | 75.0%  (25.0%-100%) | 75.0%  (25.0%-100%) | 100.0%  (66.7%-100%) | 75.0%  (25.0%-100%) | 100.0%  (66.7%-100%) |
| *Chi-square* | *–* | 5.7024 | 3.0186 | 3.6657 | 1.5580 | 2.8506 | 1.9870 | 2.6854 | 8.0059 |
| *P-value* | *–* | 0.0169 | 0.0823 | 0.0555 | 0.2120 | 0.0913 | 0.1587 | 0.1013 | 0.0047 |
| **Region** |  |  |  |  |  |  |  |  |  |
| Eastern | 64 | 85.2%  (70.4%-96.3%) | 85.7%  (71.4%-100%) | 100.0%  (50.0%-100%) | 100.0%  (50.0%-100%) | 75.0%  (50%-100%) | 100.0%  (100%-100%) | 75.0%  (75.0%-100%) | 100.0%  (100%-100%) |
| Central | 48 | 75.9%  (55.6%-88.9%) | 85.7%  (71.4%-85.7%) | 50.0%  (0.0%-100%) | 100.0%  (31.3%-100%) | 75.0%  (25.0%-100%) | 100.0%  (66.7%-100%) | 75.0%  (25.0%-100%) | 66.7%  (100%-100%) |
| Western | 50 | 68.5%  (47.2%-85.2%) | 85.7%  (57.1%-89.3%) | 50.0%  (0.0%-100%) | 62.5%  (25.0%-100%) | 75.0%  (25.0%-100%) | 100.0%  (33.3%-100%) | 75.0%  (18.8%-100%) | 100.0%  (33.3%-100%) |
| *Chi-square* | *–* | 13.5898 | 8.8835 | 4.5520 | 6.7235 | 5.3276 | 6.7870 | 7.3740 | 8.9147 |
| *P-value* | *–* | 0.0011 | 0.0118 | 0.1027 | 0.0347 | 0.0697 | 0.0336 | 0.0250 | 0.0116 |
| **Hospital level** | |  |  |  |  |  |  |  |  |
| Secondary or below | 97 | 74.1%  (55.6%-88.9%) | 85.7%  (71.4%-100%) | 50.0%  (0.0%-100%) | 75.0%  (25.0%-100%) | 75.0%  (25.0%-100%) | 100.0%  (66.7%-100%) | 75.0%  (25.0%-100%) | 100.0%  (66.7%-100%) |
| Tertiary | 65 | 85.2%  (66.7%-94.5%) | 85.7%  (71.4%-100%) | 100.0%  (0.0%-100%) | 100.0%  (25.0%-100%) | 75.0%  (50.0%-100%) | 100.0%  (100%-100%) | 75.0%  (50.0%-100%) | 100.0%  (100%-100%) |
| *Chi-square* | *–* | 6.4749 | 2.3891 | 2.2473 | 1.5830 | 2.6036 | 4.1234 | 2.5816 | 7.0312 |
| *P-value* | *–* | 0.0109 | 0.1222 | 0.1338 | 0.2083 | 0.1066 | 0.0423 | 0.1081 | 0.0080 |

## Table S3 - Sensitivity table of hospital positive response rates (PRRs).

Proportion of hospitals with a specified number of domains (rows) and overall PRR meeting or exceeding specified thresholds (columns).

|  | **PRR threshold** | | |
| --- | --- | --- | --- |
| **Number of Domains** | **≥50%** | **≥75%** | **≥100%** |
| **≥1** | 98.1% | 90.7% | 88.3% |
| **≥2** | 93.2% | 83.3% | 79.0% |
| **≥3** | 88.9% | 77.2% | 64.2% |
| **≥4** | 83.3% | 66.0% | 48.8% |
| **≥5** | 75.3% | 54.3% | 35.8% |
| **≥6** | 63.6% | 40.7% | 21.6% |
| **7** | 39.5% | 19.1% | 6.8% |
| **Overall PRR** | 81.5% | 55.6% | 6.8% |

## Table S4 - Sensitivity table of hospital positive response rates (PRRs) by hospital characteristics.

Proportion of hospitals with an overall PRR meeting or exceeding specified thresholds (columns), stratified by hospital characteristics (rows).

|  | **PRR threshold** | | |
| --- | --- | --- | --- |
| **CHARACTERISTIC** | **50.0%** | **75.0%** | **100.0%** |
| **Urban/rural** |  | | |
| Urban | 87.3% | 66.7% | 7.9% |
| Rural | 77.8% | 48.5% | 6.1% |
| **Hospital Level** |  | | |
| Secondary or below | 81.5% | 55.6% | 6.8% |
| Tertiary | 84.6% | 64.6% | 9.2% |
| **Region** |  | | |
| Eastern | 87.5% | 70.3% | 7.8% |
| Central | 83.3% | 50.0% | 6.3% |
| Western | 72.0% | 42.0% | 6.0% |

## Table S5 - Variation in positive response rate (PRR) per question by participant type, organized by domain.

PRR (abs) indicates absolute difference in PRR between principal investigator (PI) and study coordinator (SC).

| **Supportive learning environment** | | | | | **Range** | | | | **PI, Median (IQR)** | | **SC, Median (IQR)** | | **Total, Median (IQR)** | | **PI**  **PRR** | | **SC**  **PRR** | **PRR (abs)** | **Chi-square** | **P-value*** |
| --- | --- | --- | --- | --- | --- | --- | --- | --- | --- | --- | --- | --- | --- | --- | --- | --- | --- | --- | --- | --- |
| D1 | In this workgroup, people value new ideas. | | | | 1-7 | | | | 6 (5,6) | | 6 (6,6) | | 6 (5,6) | | 88.82% | | 92.90% | 4.08% | 1.58 | 0.21 |
| D4 | Differences in opinions are welcomed in this workgroup. | | | | 1-7 | | | | 6 (5,6) | | 6 (5,6) | | 6 (5,6) | | 88.20% | | 91.61% | 3.41% | 1.56 | 0.21 |
| D5 | In this workgroup, people are open to alternative ways of getting work done. | | | | 1-7 | | | | 6 (5,6) | | 6 (5,6) | | 6 (5,6) | | 85.09% | | 90.97% | 5.87% | 2.58 | 0.11 |
| D6 | People in this workgroup are eager to share information about what doesn’t work as well as to share information about what does work. | | | | 1-7 | | | | 6 (5,6) | | 6 (5,6) | | 6 (5,6) | | 81.37% | | 82.58% | 1.21% | 0.08 | 0.78 |
| D10 | If you make a mistake in this workgroup, it is often held against you. (Among clinicians taking care of patients with AMI, there is a tendency to blame individuals for errors in patient care). (*negative*) | | | | 1-5 | | | | 2 (2,3) | | 2 (2,3) | | 2 (2,3) | | 63.35% | | 66.45% | 3.10% | 0.33 | 0.56 |
| D17 | This workgroup engages in productive conflict and debate during discussions. | | | | 1-7 | | | | 5 (5,6) | | 5 (5,6) | | 5 (5,6) | | 77.02% | | 76.77% | 0.24% | <0.01 | 0.96 |
| D18 | In this workgroup, we frequently identify and discuss underlying assumptions that might affect key decisions. | | | | 1-7 | | | | 5 (5,6) | | 6 (5,6) | | 5 (5,6) | | 85.71% | | 81.29% | 4.42% | 1.12 | 0.29 |
| **Time for reflection** | | | | |  | | |  | |  | |  | |  | |  |  |  |  |  |
| D21 | There is simply no time for reflection in this workgroup. (*negative*) | | | | 1-7 | | | | 3 (2,5) | | 2 (2,4) | | 3 (2,5) | | 52.17% | | 61.29% | 9.12% | 2.67 | 0.10 |
| D22 | In this workgroup, people are too busy to invest time in improvement. (*negative*) | | | | 1-7 | | | | 2 (2,5) | | 2 (2,5) | | 2 (2,5) | | 61.49% | | 69.03% | 7.54% | 1.98 | 0.16 |
| **Leadership that reinforces learning** | | |  |  |  | |  | |  | |  | |  | |  |  |  |  |  |  |
| D23 | My manager(s) establish(es) forums for and provide(s) time and resources for identifying problems and organizational challenges. | | | | | 1-7 | | | 5 (4,6) | | 5 (4,6) | | 5 (4,6) | | 65.22% | | 74.84% | 9.62% | 3.48 | 0.06 |
| D24 | My manager(s) establish(es) forums for and provide(s) time and resources for reflecting and improving on past performance. | | | | | 1-7 | | | 5 (4,6) | | 5 (4,6) | | 5 (4,6) | | 73.29% | | 70.32% | 2.97% | 0.34 | 0.56 |
| D25 | My manager(s) listen(s) attentively. | | | | | 1-7 | | | 6 (5,6) | | 6 (5,6) | | 6 (5,6) | | 79.50% | | 79.35% | 0.15% | <0.01 | 0.97 |
| D26 | My manager(s) invite(s) input from others in discussions. | | | | | 1-7 | | | 6 (5,6) | | 6 (5,6) | | 6 (5,6) | | 79.50% | | 82.58% | 3.08% | 0.49 | 0.49 |
| **Experimentation** | |  |  | |  | |  | |  | |  | |  | |  |  |  |  |  |  |
| D27 | This workgroup experiments frequently with new product/service offerings. | | | | | 1-7 | | | 5 (4,6) | | 5 (5,6) | | 5 (4,6) | | 68.32% | | 76.77% | 8.45% | 2.83 | 0.09 |
| D28 | This workgroup experiments frequently with new ways of working. | | | | | 1-7 | | | 6 (5,6) | | 5 (5,6) | | 5 (5,6) | | 81.37% | | 83.23% | 1.86% | 0.19 | 0.67 |
| D29 | This workgroup frequently employs pilot projects or simulations when trying our new ideas. | | | | | 1-7 | | | 5 (3,5) | | 5 (3,5) | | 5 (3,5) | | 55.90% | | 57.42% | 1.52% | 0.07 | 0.79 |
| D30 | This workgroup has a formal process for conducting and evaluating experiments or new ideas. | | | | | 1-7 | | | 5 (5,6) | | 5 (5,6) | | 5 (5,6) | | 77.02% | | 78.06% | 1.05% | 0.05 | 0.82 |
| **Training** | |  |  | |  | |  | |  | |  | |  | |  |  |  |  |  |  |
| D31 | Experienced employees in this workgroup receive training when new initiatives are launched. | | | | | 1-7 | | | 6 (5,6) | | 6 (5,6) | | 6 (5,6) | | 86.34% | | 85.16% | 1.17% | 0.09 | 0.77 |
| D32 | Experienced employees in this workgroup receive training when shifting to a new position. | | | | | 1-7 | | | 6 (5,6) | | 6 (5,6) | | 6 (5,6) | | 80.75% | | 87.10% | 6.35% | 2.35 | 0.13 |
| D33 | Newly hired employees in this workgroup receive adequate training. | | | | | 1-7 | | | 6 (5,6) | | 6 (5,6) | | 6 (5,6) | | 81.37% | | 83.87% | 2.50% | 0.35 | 0.56 |
| **Knowledge acquisition** | |  |  | |  | |  | |  | |  | |  | |  |  |  |  |  |  |
| D35 | This workgroup has forums for meeting with and learning from: Experts from outside the organization. | | | | | 1-7 | | | 6 (5,6) | | 6 (5,6) | | 6 (5,6) | | 83.85% | | 85.16% | 1.31% | 0.10 | 0.75 |
| D36 | This workgroup has forums for meeting with and learning from: Experts from other departments/teams/divisions. | | | | | 1-7 | | | 6 (5,6) | | 6 (5,6) | | 6 (5,6) | | 78.26% | | 80.65% | 2.38% | 0.28 | 0.60 |
| D37 | This workgroup has forums for meeting with and learning from: Customers/clients. | | | | | 1-7 | | | 5 (3,6) | | 5 (3,6) | | 5 (3,6) | | 53.42% | | 50.97% | 2.45% | 0.19 | 0.66 |
| D38 | This workgroup regularly conducts post-audits, after-action reviews, and debriefings. | | | | | 1-7 | | | 5 (5,6) | | 5 (5,6) | | 5 (5,6) | | 75.78% | | 76.77% | 1.00% | 0.04 | 0.83 |
| **Performance monitoring** | |  |  | |  | |  | |  | |  | |  | |  |  |  |  |  |  |
| D7 | This workgroup frequently compares its performance to: Best-in-class organizations. | | | | | 1-7 | | | 6 (5,6) | | 6 (5,6) | | 6 (5,6) | | 80.12% | | 83.23% | 3.10% | 0.51 | 0.48 |
| D8 | This workgroup frequently compares its performance to: Other similar workgroups. | | | | | 1-7 | | | 6 (5,6) | | 6 (5,6) | | 6 (5,6) | | 77.02% | | 82.58% | 5.56% | 1.51 | 0.22 |
| D9 | This workgroup consistently collects information on technological trends. | | | | | 1-7 | | | 6 (6,6) | | 6 (6,7) | | 6 (6,7) | | 95.65% | | 95.48% | 0.17% | 0.01 | 0.94 |

| *AMI, acute myocardial infarction* |
| --- |

# References

1. Singer SJ, Moore SC, Meterko M, Williams S. Development of a short-form Learning Organization Survey: the LOS-27. Med Care Res Rev. 2012;69:432-59.

2. Garvin DA, Edmondson AC, Gino F. Is yours a learning organization? Harvard Bus Rev. 2008;86:109-16, 34.
