# Supplementary material for: Organizational culture in cardiovascular care in Chinese hospitals: a descriptive cross-sectional study
Source: BMC Health Serv Res. 2015 Dec 21;15:569. doi: 10.1186/s12913-015-1211-7 (PMC4685633; doi:10.1186/s12913-015-1211-7)
Supplement: Additional file 2: — Survey. China PEACE hospital survey in both English and Chinese. (PDF 427 kb) [file 12913_2015_1211_MOESM2_ESM.pdf]

CHINA PEACE-retro Hospital Survey

CHINA PEACE回顾性病例登记协作医院调查

衷心感谢您参加本研究并帮助我们了解您所在医院的情况，以及您所在医院和科室在冠心病治疗方面的模式。

本调查包括了**167**个问题，共耗时约**60**分钟。

非常感谢您的参与和宝贵的时间！

Thank you for your participation in helping us to understand the institutional characteristics, as well as the medical care patterns in your hospital and department of patients with Coronary Heart Disease.

This survey consists of 167 questions and typically takes about 60 minutes to complete.

Thank you for your time and participation.

A.	**Personal information**	填写人基本情况
A.1	Gender:<br>○ Male ○ Female	您的性别：<br>○ 男 ○ 女

A.2	Education<br>○ Junior high school<br>○ Senior high school (technical school or technical secondary school)<br>○ College (junior college)<br>○ Postgraduate	您的最高教育程度：<br>○ 初中或以下　○ 高中 (技校或中专)　○ 大学 (大专)　○ 研究生
A.3	Clinical job title:<br>○ Consultant　○ Attendant　○ Resident　○ Nurse　○ Other, please specify: ___	您目前的临床职务：<br>○ 主任或副主任医师　○ 主治医师　○ 住院医师　○ 护士　○ 其他，请说明：_____
A.4	Senior administrative position in hospital:<br>○ No　○ Yes, please specify: ___	您是否担任医院或科室管理职务？<br>○ 否　○ 是，请说明：_____
A.5	You have been working in the department for __ years.	您在目前科室已工作了 ___ 年。
**B.**	**General Information of the hospital and the department**	**医院及所在科室基本情况**
	**Instructions: This section focuses on characteristics of your hospital and department. For all questions, please reflect upon them during the 1-year period from 1/1/2011 to 12/31/2011 (for some of them, please consider 1/1/2001 to 12/31/2001, and 1/1/2006 to 12/31/2006, as specified).**<br>**Even some questions in this section might be somewhat hard to answer immediately, especially those about the characteristics of your hospital or department in 2001 and 2006. Please try best to find the answer - as accurate as possible - to every applicable question.**	**填写说明：这部分主要希望了解您所在医院或科室的一般情况。对于本部分的问题，请通过回忆 2011 年（2011 年 1 月 1 日至 12 月 31 日）的情况填写（对于个别问题请按照特殊说明根据 2001 年（2001 年 1 月 1 日至 12 月 31 日）和 2006 年（2006 年 1 月 1 日至 12 月 31 日）的情况填写）。**<br>**这部分中的部分问题可能对您来说很难立刻明确回答，特别是一些调查的是您所在医院或科室的 2001 年和 2006 年的情况。但我们非常希望您能尽力找到每一个问题的准确答案。**
B.1	Affiliated hospital of medical college:<br>○ No　○ Yes, please specify the name of the college: _________ **[Skip to B3]**	您所在的医院是否医学院附属医院？<br>○ 否　○ 是，请说明医学院名称：_________ **[转至B3]**
B.2	Teaching hospital of medical college:<br>○ No　○ Yes, please specify the name of the college: _________	您所在的是否医学院教学医院？<br>○ 否　○ 是，请说明医学院名称：_________
	Total No. in your department	您所在科室以下指标的各年总数（非必填）

		In 2001	In 2006	In 2011		2001年	2006年	2011年
B.3	Beds				床位			
B.4	Consultants				主任或副主任医师			
B.5	Attendants				主治医师			
B.6	Residents				住院医师			
B.7	Nurses				护士			

B.8	Is there any other department in your hospital providing inpatient treatment for AMI?<br>o No o Yes, please specify the name of the department: _________	您所在的医院是否还有其它科室也收治AMI患者？<br>o 否 o 是，科室名称为：____________________
B.9	Coronary Care Unit (CCU) in hospital?<br>o No o Yes, please specify the No. of beds: _________	您所在的医院是否设有冠心病监护病房（CCU）？<br>o 否 o 是，床位数为：____________________
B.10	Cath lab in hospital?<br>o No **[Skip to B12]** o Yes, please specify when started: _________	您所在的医院是否设有心脏导管室？<br>o 否 **[转至B12]** o 是，开始年份为：____________________
B.11	How many qualified cardiac interventionalist there are in your hospital: _________ o unknown	您所在的医院有多少名有资质的心脏介入医生：______ o 不清楚
B.12	Could CABG be performed in hospital?<br>o No o Yes, please specify the No. of cases in 2011: _______	您所在的医院是否能开展心脏搭桥手术？<br>o 否 o 是，2011年的例数为：______
B.13	Independent emergency department?<br>o No o Yes, please specify the No. of cardiologists in charge in emergency department normally: _______	您所在的医院是否设有急诊？<br>o 否 o 是，急诊通常的值班心内科医生人数为：______
B.14	Formal GCP training of clinical staff in your department?<br>o No o Yes o Unknown	您所在的贵科室是否有医务人员参加过GCP的系统培训？<br>o 否 o 是 o 不清楚
B.15	Have your apartment participated in international clinical trials?<br>o No o Yes, please specify the names of the trials: _______ o Unknown	您所在的贵科室是否参加过国际大规模临床试验？<br>o 否 o 是，研究名称为：_____________ o 不清楚

B.16 SFDA certified site for CVD drug trials?
o No o Yes o Unknown

您所在的医院是否设有SFDA批准的心血管病临床药理基地？
o 否 o 是 o 不清楚

B.17 Existence of Ethics Committee in hospital?
o No o Yes o Unknown

您所在的医院是否设有专门的伦理委员会？
o 否 o 是 o 不清楚

Total No. in your hospital

您所在医院以下指标的各年总数（非必填）

		**In 2001**	**In 2006**	**In 2011**		**2001年**	**2006年**	**2011年**
B.18	Patients with stroke				脑卒中住院患者			
B.19	Patients with ischemic stroke				缺血性脑卒中住院患者			
B.20	Patients with hemorrhagic stroke				出血性脑卒中住院患者			

B.21 Independent neurology department?
o No o Yes, please specify the No. of beds in the department: ______

您所在的医院是否设有专门的神经科？
o 否 o 是，神经科目前的床位数为：___________________

B.22 Carotid endarterectomy performed in hospital?
o No o Yes, please specify when started: ______ o Unknown

您所在的医院内可以实施颈动脉内膜切除术？
o 否 o 是，开始年份为：___________________ o 不清楚

B.23 Carotid stenting performed in hospital?
o No o Yes, please specify when started: ______ o Unknown

您所在的医院内可以实施颈动脉内支架置入术？
o 否 o 是，开始年份为：___________________ o 不清楚

The average cost of the following items in your hospital

您所在的医院以下检测项目的大致费用（非必填）

	**Items**	**Cost, ￥**	**项目**	**花费/元**
B.24	Biochemical test, including glucose, lipid, liver function, renal function, CRP or hsCRP		血生化检测，包括血糖、血脂、肝功能、肾功能，以及CRP 或 hsCRP	
B.25	Coagulation function test		凝血功能检测	
B.26	BNP or NT-proBNP		BNP 或 NT-proBNP	
B.27	Stress test		负荷试验	
B.28	UCG		心脏超声	

B.29	Cardiac CT		冠脉CT	
B.30	Carotid US		颈动脉超声	

C.	**Diagnosis and treatment for CHD**	**冠心病的诊疗**
	**Instructions: This section focuses on hospital processes and care of patients with AMI. For all questions, please reflect upon them during the 1-year period from 1/1/2011 to 12/31/2011.**	**填写说明：这部分主要希望了解您所在医院或科室对 AMI 患者的诊断治疗情况。对于本部分的问题，请通过回忆 2011 年（2011 年 1 月 1 日至 12 月 31 日）的情况填写。**
C.1	Routine diagnostic test of CK for ACS patients after admission?<br>o No o Yes, please specify the average time delay in reporting results: _______ o Unknown	ACS患者入院常规检测肌酸激酶（CK）？<br>o 否 o 是，通常平均需要等____小时可以得到结果 o 不清楚
C.2	Routine diagnostic test of CK-MB for ACS patients after admission?<br>o No o Yes, please specify the average time delay in reporting results: _______ o Unknown	ACS患者入院常规检测肌酸激酶同工酶（CK-MB）？<br>o 否 o 是，通常平均需要等____小时可以得到结果 o 不清楚
C.3	Routine diagnostic test of troponin for ACS patients after admission?<br>o No o Yes, please specify the average time delay in reporting results: _______ o Unknown	ACS患者入院常规检测肌钙蛋白（troponin）？<br>o 否 o 是，通常平均需要等____小时可以得到结果 o 不清楚
C.4	Are patients who are stable after PCI admitted to an intensive care unit? SAMI-Q25<br>o Always o Usually o Sometimes o Rarely o Unknown	您所在的医院行PCI后情况稳定的患者会转入ICU或CCU吗？<br>o 总是 o 通常 o 有时候 o 很少 o 不清楚
C.5	Did your emergency department use a uniform protocol to care for patients who arrived to the emergency department with **STEMI**? SAMI-Q26<br>o No o Yes o Unknown	您所在的医院急诊科在处理来急诊就诊的**STEMI**患者时，有统一的方案或流程吗？<br>o 否 o 是 o 不清楚

C.6	Did your emergency department use a uniform protocol to care for patients who arrived to the emergency department with **Unstable Angina/NSTEMI**? SAMI-Q27<br>o No o Yes o Unknown	您所在的医院急诊科在处理来急诊就诊的**NSTEMI或不稳定心绞痛**患者时，有统一的方案或流程吗？<br>o 否 o 是 o 不清楚
C.7	Did your hospital use simulations (i.e., trial exercises, dry-runs) to practice any of the following AMI care processes? ***[Check all that apply]*** SAMI-Q28<br>□ Door-to-balloon or door-to-drug protocols<br>□ Chest pain in hospitalized patients<br>□ Inpatient codes (e.g., cardiac arrest, respiratory failure)<br>□ None above<br>□ Unknown	对以下AMI院内诊疗方案，您所在的医院会组织模拟演练进行练习吗？***[可多选]***<br>□ 进门到再灌注治疗诊疗方案<br>□ 胸痛患者诊疗方案<br>□ 心脏骤停、呼吸衰竭等患者复苏方案<br>□ 以上都没有<br>□ 不清楚
C.8	To which patient care unit were patients who were stable with Unstable Angina/NSTEMI most likely admitted? SAMI-Q29<br>o CCU o ICU o Step-down unit o Designated chest pain/telemetry/cardiology floor o General medicine floor o We did not have a routine method of assigning beds for patients with Unstable Angina/NSTEMI o Unknown	如果**NSTEMI或不稳定心绞痛**患者病情稳定后，一般会到什么病区？<br>o CCU o ICU o 过渡监护治疗病房 o 特定的胸痛过渡监护病房 o 普通内科病房 o 不固定 o 不清楚
C.9	Did all, or nearly all, patients with AMI have a cardiologist as their primary attending physician? SAMI-Q30<br>o No o Yes **[Skip to C11]** o Unknown	您所在的医院治疗的绝大多数AMI患者的主管医生都是心内科专业的医生吗？<br>o 否 o 是 **[转至C11]** o 不清楚
C.10	Were cardiology consults required for all patients with AMI? SAMI-Q30a<br>o No o Yes o Unknown	您所在的医院治疗的AMI患者都需要有心内科专业会诊吗？<br>o 否 o 是 o 不清楚

C.11	In the intensive care unit, who was primarily responsible for the care of patients with AMI? ***[Check all that apply]*** SAMI-Q31<br>□ Critical care physicians (i.e., intensivists)<br>□ Cardiologist/s based exclusively in the unit<br>□ Other cardiologists<br>□ Other, please specify: ______<br>□ Unknown	在ICU或CCU，由谁来主要负责AMI患者的诊疗？***[可多选]***<br>□ 重症监护专业医生<br>□ ICU或CCU的专门心内科专业医生<br>□ 其他心内科专业医生<br>□ 其他专业医生，请说明：______<br>□ 不清楚
C.12	Electronic medical record?<br>o No **[Skip to C14]** o Yes, please specify when started: ______<br>o Unknown	您所在的医院是否使用电子化病历？<br>o 否 **[转至C14]** o 是，开始年份为：__________________ o 不清楚
C.13	Did your hospital use an electronic medical record (EMR) in the following areas? ***[Check all that apply]*** SAMI-Q34<br>□ Emergency department<br>□ Inpatient floors<br>□ Critical care units<br>□ Affiliated ambulatory offices/clinics<br>□ None above	您所在的医院在下列各部门是否使用电子病历？***[可多选]***<br>□ 急诊科<br>□ 住院病房<br>□ ICU或CCU<br>□ 本院所辖急救中心<br>□ 以上都没有

C.14	On the inpatient floors, did your hospital have the following electronic capabilities? ***[Check all that apply]*** SAMI-Q35<br>□ Computerized assisted physician order entry<br>□ Computer prompts to alert user to potential drug-drug interactions or allergies<br>□ Computer prompts to alert user to potential errors in dosing and information<br>□ Computer prompts to alert user to medication order expiration<br>□ Computer prompts to improve adherence to core measures for AMI care (e.g., beta-blocker use)<br>□ None above	在您所在的医院的住院病房中，是否有以下电子化辅助功能？***[可多选]***<br>□ 计算机辅助医嘱录入<br>□ 计算机辅助提醒不同药物的交互作用，以及药物过敏信息<br>□ 计算机辅助提醒用药剂量等信息方面可能的错误<br>□ 计算机辅助提醒用药医嘱到期<br>□ 计算机辅助提醒依照AMI核心治疗措施治疗（如指南推荐的使用β受体阻滞剂）<br>□ 以上都没有
C.15	In the emergency department, were prior ECG's electronically available at the time of care? SAMI-Q36<br>o No o Yes o Unknown	您所在的医院急诊科的心电图是否以电子化形式保存，以供之后查阅？<br>o 否 o 是 o 不清楚
C.16	Did physicians regularly use explicit protocols or clinical pathways for patients with AMI? SAMI-Q37<br>o No o Yes o Unknown	您所在的医院医生是否常规根据详细的临床诊疗路径或方案管理AMI患者？<br>o 否 o 是 o 不清楚
C.17	Did clinicians on the inpatient care units regularly use order sets (either paper-based or electronic) for patients with STEMI? SAMI-Q38<br>o No o Yes o Unknown	您所在的医院住院病房的医生是否经常使用采用纸质或电子化的医嘱套餐管理STEMI患者？<br>o 否 o 是 o 不清楚
C.18	Did clinicians on the inpatient care units regularly use order sets (either paper-based or electronic) for with Unstable Angina/NSTEMI? SAMI-Q39<br>o No o Yes o Unknown	您所在的医院住院病房的医生是否经常使用采用纸质或电子化的医嘱套餐管理NSTEMI或不稳定心绞痛患者？<br>o 否 o 是 o 不清楚

C.19 Which of the following types of physicians were at the hospital 24-hours/day and 7-days/week? ***[Check all that apply]*** SAMI-Q42<br>□ Critical care physicians (i.e., intensivists)<br>□ Non-interventional cardiologists<br>□ Interventional cardiologists<br>□ Cardiology fellows (including non-interventional and interventional)<br>□ Hospitalists<br>□ None above	您所在的医院，有哪些不同类型的医生会7×24小时在院值班？***[可多选]***<br>□ 重症监护医生<br>□ 非介入的心内科医生<br>□ 介入医生<br>□ 心内科住院医生（无论是否介入治疗方向）<br>□ 普通住院病房医生<br>□ 以上都没有
C.20 Are there any protocols used to guide nurses on when to call the attending cardiologist for patients with AMI? SAMI-Q43<br>○ No ○ Yes ○ Unknown	您所在的医院是否为护士准备了专门的指导方案——告诉他们在AMI患者出现什么情况下应该通知主管医生？<br>○ 否 ○ 是 ○ 不清楚
C.21 Patients with acute coronary syndrome who arrived by Emergency medical service (ambulance):<br>○ None **[Skip to C25]** ○ 1–25% ○ 26–50% ○ 51–75% ○ 76-100% ○ Unknown	到贵院就诊的ACS患者中，有多少是急救中心（救护车）送来的？<br>○ 没有 **[转至C25]** ○ 1–25% ○ 26–50% ○ 51–75% ○ 76-100% ○ 不清楚
C.22 Emergency medical service routinely gives pre-alert calls?<br>○ No ○ Yes ○ Unknown	急救中心通常会在患者未抵达医院前打电话通知您所在的医院吗？<br>○ 否 ○ 是 ○ 不清楚
C.23 Patients with acute coronary syndrome who undergo ECG en route to hospital:<br>○ None ○ 1–25% ○ 26–50% ○ 51–75% ○ 76-100% ○ Unknown	有多少ACS患者在到达医院的途中完成了ECG检查？<br>○ 没有 ○ 1–25% ○ 26–50% ○ 51–75% ○ 76-100% ○ 不清楚
C.24 Emergency medical service routinely tell your hospital the results of ECG?<br>○ No ○ Yes ○ Unknown	急救中心通常会在患者未抵达医院前通知医院患者的ECG结果吗？<br>○ 否 ○ 是 ○ 不清楚

C.25 Formal training of triage staff for assessing acute coronary syndrome?<br>o No o Yes o Unknown	您所在的医院是否为导医人员提供针对ACS评估的正式培训？<br>o 否 o 是 o 不清楚
C.26 Dedicated space in triage area for immediate ECG?<br>o No o Yes o Unknown	您所在的医院的导医区是否有专门的空间用于快速ECG检查？<br>o 否 o 是 o 不清楚
C.27 Written criteria for immediate ECG in emergency department?<br>o No o Yes o Unknown	您所在的医院是否对急诊科的快速ECG检查有成文的标准和要求？<br>o 否 o 是 o 不清楚
C.28 Expected interval between patients' arriving and ECG?<br>o ≤5 min o 6–20 min o >20 min o No expected time o Unknown	您所在的医院对从患者来到医院，到完成快速心电图检测的时间间隔的要求是？<br>o ≤5 min o 6–20 min o >20 min o 无时间要求 o 不清楚
C.29 Dedicated ECG technicians in emergency department?<br>o No o Yes, only some shifts o Yes, always o Unknown	您所在的医院中急诊科是否设有专门的ECG技术人员？<br>o 否 o 是，尽在一些班次有 o 是，一直有 o 不清楚
C.30 Thrombolysis for AMI patients in hospital?<br>o No **[Skip to C38]** o Yes, please specify when started: ______	您所在的医院是否可以对AMI患者实施溶栓治疗？<br>o 否 **[转至C38]** o 是，开始年份为：____________________
C.31 Does your hospital have a set protocol to identify eligible patients for thrombolysis?<br>o No o Yes o Unknown	您所在的医院是否有用于筛选溶栓治疗合格患者的固定方案流程？<br>o 否 o 是 o 不清楚
C.32 Does your hospital have a set protocol to assess contraindications of thrombolysis?<br>o No o Yes o Unknown	您所在的医院是否有用于排除溶栓治疗禁忌症的固定方案流程？<br>o 否 o 是 o 不清楚

C.33	Who makes the decision about thrombolysis in your hospital?<br>o Emergency medicine physician alone<br>o Emergency medicine physician with a cardiac consultation<br>o Only Cardiologist<br>o Unknown	您所在的医院中谁负责决定给患者进行溶栓治疗？<br>o 可以有急诊科医生单独决定<br>o 急诊科医生需要心内科会诊意见才能决定<br>o 仅有心内科医生可以决定<br>o 不清楚
C.34	In your hospital, where do patients with AMI receive thrombolysis?<br>o In the emergency department<br>o In the cardiology department (or general medicine department)<br>o In the ICU or CCU<br>o Unknown	您所在的医院中，AMI患者在哪里接受溶栓治疗？<br>o 在急诊科<br>o 在心内科（或普通内科）病房<br>o 在ICU或CCU<br>〇 不清楚
C.35	Where are the thrombolytic medicines stored and prepared?<br>o Stored and prepared in the department where thrombolysis is done<br>o Prepared in the department where thrombolysis is done, but stored in another location<br>o Stored and prepared in some location other than the department where thrombolysis is done<br>o Unknown	您所在的医院中，溶栓药物在哪里保存和配制？<br>o 在进行溶栓治疗的科室中保存和配制<br>o 在进行溶栓治疗的科室中配制，但在其他地方保存<br>o 在进行溶栓治疗的科室以往的地方保存和配制<br>o 不清楚
C.36	Informed Consent before thrombolysis?<br>o Not necessary<br>o Only orally obtained informed consent is needed<br>o One written informed consent form is needed<br>o More than one written informed consent form is needed<br>o Unknown	患者在接受溶栓治疗前的知情同意过程？<br>o 不需要<br>o 只需要口头知情同意<br>o 需要1份书面知情同意书<br>o 需要1份以上的书面知情同意书<br>o 不清楚

C.37	Prepayment before thrombolysis?<br>○ No<br>○ Yes, please specify the average amount approximately: ___ ("-1" if unknown)<br>○ Unknown	患者在接受溶栓治疗前必须预先垫付医疗费？<br>○ 否<br>○ 是，预付的平均金额大约为：___ （如果不清楚请填"-1"）<br>○ 不清楚
C.38	Primary PCI was performed in your hospital for STEMI patients?<br>○ No **[Skip to C60]** ○ Yes, please specify when started: ___	您所在的医院是否可以对STEMI患者实施急诊PCI治疗？<br>○ 否 **[转至C60]** ○ 是，开始年份为：___________________
C.39	Activation of catheterization laboratory on weekdays?<br>○ Emergency medicine physician with cardiologist<br>○ Cardiologist alone<br>○ Emergency medicine physician alone<br>○ Unknown	在工作日的工作时间，由谁决定可以让导管室准备PCI？<br>○ 需要急诊科医生和心内科医生一起决定<br>○ 可以由心内科医生单独决定<br>○ 可以由急诊科医生单独决定<br>○ 不清楚
C.40	Activation of catheterization laboratory at night and on weekends?<br>○ Emergency medicine physician with cardiologist<br>○ Cardiologist alone<br>○ Emergency medicine physician alone<br>○ Unknown	在周末或晚上，由谁决定可以让导管室准备PCI？<br>○ 需要急诊科医生和心内科医生一起决定<br>○ 可以由心内科医生单独决定<br>○ 可以由急诊科医生单独决定<br>○ 不清楚

C.41	Process for activating catheterization team?<br>○ After communicating with the emergency department, interventional cardiologist activates catheterization laboratory by calling staff or a central page operator<br>○ Emergency department makes at least two calls: one to the interventional cardiologist and another to a central page operator, who pages catheterization laboratory staff<br>○ Emergency department makes a single call to a central page operator, who then pages interventional cardiologist and catheterization laboratory staff<br>○ No standard approach<br>○ Other<br>○ Unknown	您所在的医院中，要求导管室准备PCI的常规程序？<br>○ 急诊科医生与介入医生沟通后，介入医生打电话给导管室安排手术<br>○ 急诊科医生至少打两个电话，一个给介入医生，另一个给导管室值班总机，导管室值班总机通知导管室其他人员<br>○ 急诊科医生打一个电话给导管室值班总机，导管室值班总机安排介入医生和其他人员<br>○ 没有标准化程序<br>○ 其他<br>○ 不清楚
C.42	Activation of on-call staff for catheterization laboratory?<br>○ Page operator is not used<br>○ Page operator is used; confirmation of page receipt is required<br>○ Page operator is used; no confirmation of page receipt is required<br>○ No standard approach<br>○ Unknown	您所在的医院中，要求导管室值班人员准备手术的常规程序？<br>○ 不需要导管室值班总机<br>○ 需要导管室值班总机，而且需要确认手术安排计划<br>○ 需要导管室值班总机，但不需要确认手术安排计划<br>○ 没有标准化程序<br>○ 不清楚
C.43	First physician notified after STEMI diagnosis in emergency department?<br>○ Cardiologist ○ Interventional cardiologist ○ Patient’s primary care physician ○ Other or variable ○ Unknown	您所在的医院中，急诊室中确诊STEMI后第一个通知的医生是？<br>○ 心内科医生 ○ 介入医生 ○ 全科医生 ○ 其他或不一定<br>○ 不清楚

C.44	Laboratory and radiographic results are needed to activate catheterization laboratory?<br>o Yes o No o No standard approach o Unknown	您所在的医院中，在要求导管室准备PCI前，是否必须有实验室或影像学检查报告？<br>o 是 o 否 o 没有标准化程序 o 不清楚
C.45	Process after emergency medical service transmits ECG results?<br>o Emergency department waits for patient to arrive at hospital to determine whether catheterization laboratory should be activated<br>o Emergency department contacts cardiologist while the patient is en route to determine whether catheterization laboratory should be activated<br>o Emergency department activates catheterization laboratory while the patient is still en route to the hospital<br>o No standard approach or variable approach<br>o Not applicable because ECG data not transmitted en route<br>o Not applicable because ECG never performed en route<br>o Unknown	医院从急救中心收到其发送的ECG结果之后的程序？<br>o 急诊科等待患者到院后决定是否通知导管室准备<br>o 急诊科在患者到院前联系心内科决定是否通知导管室准备<br>o 急诊科在患者到院前直接通知导管室准备<br>o 没有标准程序或有多种方法<br>o 因为ECG结果不会在来院途中送至医院，所以不适用<br>o 因为来院途中不会做ECG检查，所以不适用<br>o 不清楚
C.46	Expected interval between page and arrival of staff in catheterization laboratory?<br>o ≤20 min o 21–30 min o >30 min o No expected time o Unknown	您所在的医院对从安排手术，到导管室人员就位的时间间隔的要求是？<br>o ≤20 min o 21–30 min o >30 min o 无时间要求 o 不清楚
C.47	Expected interval between page and arrival of interventional cardiologist?<br>o ≤20 min o 21–30 min o >30 min o No expected time o Unknown	您所在的医院对从安排手术，到介入医生就位的时间间隔的要求是？<br>o ≤20 min o 21–30 min o >30 min o 无时间要求 o 不清楚

C.48	Someone is always available to transport patients from emergency department to catheterization laboratory?<br>o No o Yes o Unknown	您所在的医院中是否任何时候都有人可以负责将病人从急诊科转移到导管室？<br>o 否 o 是 o 不清楚
C.49	Initiation of patient transport from emergency department to catheterization laboratory?<br>o After catheterization laboratory notifies emergency department it is ready<br>o A set interval after the decision is made regarding PCI<br>o No standard approach<br>o Other approach<br>o Unknown	您所在的医院中需要怎样的步骤才能决定开始将需行PCI的病人从急诊科转移至导管室？<br>o 等导管室通知急诊科他们已经准备好了<br>o 在通知导管室决定将行PCI之后的一段固定时间之后（如通知导管室15分钟后开始转移病人）<br>o 无标准化程序<br>o 其他程序<br>o 不清楚
C.50	Minimum number of nurses and technicians required in catheterization laboratory before patient is transported from emergency department?<br>o Interventional cardiologist must be present<br>o Interventional cardiologist may not be present but need presence of 1 staff person<br>o Interventional cardiologist may not be present but need presence of 2-4 staff person<br>o No set number<br>o Unknown	在从急诊科转移病人之前，导管室至少需要多少护士和技术人员？<br>o 介入医生必须在场<br>o 介入医生可以不在场，但必须有1位其他工作人员在场<br>o 介入医生可以不在场，但必须有2-4位其他工作人员在场<br>o 没有固定人员数量要求<br>o 不清楚
C.51	Elective catheterization cases rescheduled for emergency PCI?<br>o Yes o No o It depends o Unknown	您所在的医院是否会因为急诊PCI而调整其他择期PCI的时间安排？<br>o 是 o 否 o 视情况而定 o 不清楚

C.52	If interventionalist is present, number of staff required to begin PCI?<br>o 1 o 2 o 3 o 4 o Unknown	如果介入医生在场，导管室还需要几位人员才可以开始PCI？<br>o 1 o 2 o 3 o 4 o 不清楚
C.53	Catheterization laboratory is left so that next PCI can begin promptly?<br>o Yes o No o No standard policy o Unknown	PCI手术结束后，是否需要将导管室保持在一定状态下，以便下一台手术可以尽快开始？<br>o 是 o 否 o 没有标准规定 o 不清楚
C.54	Cardiology fellows participate in performing PCI?<br>o No o Yes o Unknown	心内科医生是否会参与PCI手术？<br>o 否 o 是 o 不清楚
C.55	Staff in critical care area are routinely cross-trained to cover catheterization laboratory?<br>o No o Yes o Unknown	ICU或CCU的工作人员是否会常规接受导管室工作的相关培训吗？<br>o 否 o 是 o 不清楚
C.56	Location of catheterization laboratory?<br>o Elevator required to travel from emergency department<br>o Same floor as emergency department	您所在的医院中导管室位于什么位置？<br>o 与急诊科在不同楼层，需要经乘电梯才能到达<br>o 与急诊科在同一楼层
C.57	An attending cardiologist is always at the hospital?<br>o No o Yes o Unknown	您所在的医院中是否始终有至少一位心内科主治医生值班<br>o 否 o 是 o 不清楚
C.58	Informed Consent before primary PCI?<br>o Not necessary<br>o Only orally obtained informed consent is needed<br>o One written informed consent form is needed<br>o More than one written informed consent form is needed<br>o Unknown	患者在接受急诊PCI前的知情同意过程？<br>o 不需要<br>o 只需要口头知情同意<br>o 需要1份书面知情同意书<br>o 需要1份以上的书面知情同意书<br>o 不清楚

C.59	Prepayment before primary PCI?<br>○ No<br>○ Yes, please specify the average amount approximately ___ ("-1" if unknown)<br>○ Unknown	患者在接受急诊PCI前必须预先垫付医疗费？<br>○ 否<br>○ 是，预付的平均金额大约为___ （如果不清楚请填"-1"）<br>○ 不清楚
C.60	Does your hospital measure the following time intervals? ***[Check all that apply]***<br>□ Door to ECG<br>□ Door to needle<br>□ Door to balloon<br>□ None above<br>□ Unknown	您所在的医院会记录以下的治疗时间延误长度吗？ ***[可多选]***<br>□ 从就诊到完成ECG<br>□ 从就诊到执行溶栓治疗<br>□ 从就诊到急诊PCI球囊扩张<br>□ 以上都没有<br>□ 不清楚
C.61	Do your hospital feedback the time intervals to someone? ***[Check all that apply]***<br>□ No<br>□ Yes, to physician staff involved in the care<br>□ Yes, to nursing staff involved in the care<br>□ Yes, to pharmacy staff involved in the care<br>□ Yes, to other staff involved in the care<br>□ Unknown	您所在的医院会将记录的治疗时间延误长度反馈给有关人员吗？ ***[可多选]***<br>□ 不会<br>□ 会反馈给相关的医生<br>□ 会反馈给相关的护士<br>□ 会反馈给相关的药剂师<br>□ 会反馈给相关的其他人员<br>□ 不清楚

C.62	Do your hospital report the analyze results about the time intervals regularly? ***[Check all that apply]***<br>□ No<br>□ Yes, to departments involved in the care (the emergency department, the cardiology department)<br>□ Yes, to other department in your hospital<br>□ Yes, to other institutions outside your hospital<br>□ Unknown	您所在的医院会定期将治疗时间延误的分析结果报告给有关单位吗？***[可多选]***<br>□ 不会<br>□ 会将分析报告发给相关科室（如急诊科、心内科）<br>□ 会将分析报告发给本院的其他科室<br>□ 会将分析报告发给院外的其他单位<br>□ 不清楚
D.	**Organizational learning characteristics**	组织学习特征

	**Instructions: This section focuses on the organizational learning and measurements to improve AMI care, including supportive environment and leadership, experimentation and training, knowledge acquisition, reflection and performance monitoring, etc. Please draw on your own experiences in your current role working with clinical staff and administration. For all questions, please reflect upon them during the 1-year period from 1/1/2011 to 12/31/2011.**<br>**Although some questions in this section look similar, there are differences between them and you should treat each one as a separate question. The best approach is to answer each question fairly quickly. That is, don't try to count up the number of times you felt a particular way, but rather indicate the alternative that seems most reasonable.**<br>**The definition of "workgroup" below is the department, unit, ward, or group caring AMI patients that you are working at.**<br>**This section adopts 7-point (from highly inaccurate to highly accurate). If you think the options are difficult to understand or distinguish, please grade the accuracy here using actual numbers, while 1 is the lowest (highly inaccurate), 7 is the highest (highly accurate), then choose the corresponding option.**	填写说明：这部分主要希望了解您所在的工作团队为改善 **AMI** 患者的诊断治疗而进行的组织学习的情况，包括环境与领导支持、试验与培训、相关知识的交流与获取、绩效监督与反思等方面。对于本部分的问题，请通过回忆 **2011** 年（**2011** 年 **1** 月 **1** 日至 **12** 月 **31** 日）的情况填写，判断题干中的情况在您所在工作团队中出现的频率，或用于描述您所在工作团队状态时的准确程度。<br>尽管这部分中的一些问题看起来类似，但他们之间都各有不同。您应把他们中的每一个都作为独立的问题来回答。因此回答这些部分问题最好的办法就是快速选出您认为合适的选项，而不用在意哪些问题相似。<br>"工作团队"是指您所在的为 **AMI** 患者提供治疗的科室、临床单元、病房，或工作小组。<br>在这部分的问题中，采用了针对准确程度的 **7** 分类选项（从"很不准确"到"很准确"）。如果您认为所给的选项不容易理解或难以区别时，可以试着对这个问题的准确程度打分，最低给 **1** 分，最高给 **7** 分，您觉得应打多少分，然后选择对应选项。
D.1	In this workgroup, people value new ideas.<br>○ highly inaccurate ○ inaccurate ○ somewhat inaccurate ○ not sure ○ somewhat accurate ○ accurate ○ highly accurate	您所在的工作团队中，大家都很重视新的想法和观点。<br>○ 很不准确 ○ 不准确 ○ 部分不准确 ○ 不确定 ○ 部分准确 ○ 准确 ○ 很准确
D.2	Clinicians are encouraged to creatively solve problems related to AMI care processes. (60)<br>○ Never ○ Rarely ○ Sometimes ○ Usually ○ Always	您所在的团队鼓励临床医生去创造性的解决 AMI 诊疗过程中的相关问题。<br>○ 从不 ○ 很少 ○ 有时 ○ 经常 ○ 总是
D.3	Innovative ideas about AMI care are shared widely in the hospital. (61)<br>○ Never ○ Rarely ○ Sometimes ○ Usually ○ Always	在医院内，大家会广泛分享关于 AMI 诊疗的新想法和新观点。<br>○ 从不 ○ 很少 ○ 有时 ○ 经常 ○ 总是

D.4	Differences in opinions are welcomed in this workgroup.<br>○ highly inaccurate ○ inaccurate ○ somewhat inaccurate ○ not sure ○ somewhat accurate ○ accurate ○ highly accurate	您所在的工作团队欢迎不同的意见。<br>○ 很不准确 ○ 不准确 ○ 部分不准确 ○ 不确定 ○ 部分准确 ○ 准确 ○ 很准确
D.5	In this workgroup, people are open to alternative ways of getting work done.<br>○ highly inaccurate ○ inaccurate ○ somewhat inaccurate ○ not sure ○ somewhat accurate ○ accurate ○ highly accurate	您所在的工作团队中，大家对用不同的工作方法持开放包容的态度。<br>○ 很不准确的 ○ 不准确 ○ 部分不准确 ○ 不确定 ○ 部分准确 ○ 准确 ○ 很准确的
D.6	People in this workgroup are eager to share information about what doesn't work as well as to share information about what does work.<br>○ highly inaccurate ○ inaccurate ○ somewhat inaccurate ○ not sure ○ somewhat accurate ○ accurate ○ highly accurate	与分享某种有效的方法的信息一样，您所在的工作团队的成员同样渴望分享关于某种无效的方法的信息。<br>○ 很不准确 ○ 不准确 ○ 部分不准确 ○ 不确定 ○ 部分准确 ○ 准确 ○ 很准确
D.7	This workgroup frequently compares its performance to: Best-in-class organizations.<br>○ highly inaccurate ○ inaccurate ○ somewhat inaccurate ○ not sure ○ somewhat accurate ○ accurate ○ highly accurate	您所在的工作团队经常将自己的表现与同行中最好的工作团队进行比较。<br>○ 很不准确 ○ 不准确 ○ 部分不准确 ○ 不确定 ○ 部分准确 ○ 准确 ○ 很准确
D.8	This workgroup frequently compares its performance to: Other similar workgroups.<br>○ highly inaccurate ○ inaccurate ○ somewhat inaccurate ○ not sure ○ somewhat accurate ○ accurate ○ highly accurate	您所在的工作团队经常将自己的表现与其他类似的工作团队进行比较。<br>○ 很不准确 ○ 不准确 ○ 部分不准确 ○ 不确定 ○ 部分准确 ○ 准确 ○ 很准确
D.9	This workgroup consistently collects information on technological trends.<br>○ highly inaccurate ○ inaccurate ○ somewhat inaccurate ○ not sure ○ somewhat accurate ○ accurate ○ highly accurate	您所在的工作团队会持续的关注本领域诊疗技术发展动态的相关信息。<br>○ 很不准确 ○ 不准确 ○ 部分不准确 ○ 不确定 ○ 部分准确 ○ 准确 ○ 很准确

D.10 If you make a mistake in this workgroup, it is often held against you. (Among clinicians taking care of patients with AMI, there is a tendency to blame individuals for errors in patient care). (66)<br>○ Never ○ Rarely ○ Sometimes ○ Usually ○ Always	如果您在工作中出现失误，工作团队会因此责怪您（例如在照顾 AMI 患者的临床医生中，他们倾向于将患者诊疗过程中的错误归咎于个人）。<br>○ 从不 ○ 很少 ○ 有时 ○ 经常 ○ 总是
D.11 Clinicians caring for patients with AMI are easily able to address problems and tough issues with their department heads/chiefs. (56)<br>○ Never ○ Rarely ○ Sometimes ○ Usually ○ Always	AMI 患者的主管医生可以与其科室主任之间一起解决某些棘手问题。<br>○ 从不 ○ 很少 ○ 有时 ○ 经常 ○ 总是
D.12 Department heads/chiefs are easily able to address problems and tough issues with senior level administration.(57)<br>○ Never ○ Rarely ○ Sometimes ○ Usually ○ Always	科室主任可以能与医院管理层一起解决棘手问题。<br>○ 从不 ○ 很少 ○ 有时 ○ 经常 ○ 总是
D.13 Nurses are comfortable checking with physicians if they have concerns about patient care.(65)<br>○ Never ○ Rarely ○ Sometimes ○ Usually ○ Always	如果护士对患者诊疗存在疑虑，他们可以与医生确认，而无需有任何顾虑。<br>○ 从不 ○ 很少 ○ 有时 ○ 经常 ○ 总是
D.14 Clinicians involved in the care of patients with AMI value each others' skills and talents (e.g., physicians value nurses' skills and talents and vice-versa).(58)<br>○ Never ○ Rarely ○ Sometimes ○ Usually ○ Always	在参与 AMI 患者治疗的临床人员中，他们互相尊重他人的技术与能力（比如，医生尊重护士的技术和能力，反之亦然）。<br>○ 从不 ○ 很少 ○ 有时 ○ 经常 ○ 总是
D.15 Clinicians involved in the care of patients with AMI avoid sharing responsibility for medical errors. ○ Never ○ Rarely ○ Sometimes ○ Usually ○ Always. (59)<br>○ Never ○ Rarely ○ Sometimes ○ Usually ○ Always	参与 AMI 患者治疗的临床医生会不愿共同分担医疗错误的责任。<br>○ 从不 ○ 很少 ○ 有时 ○ 经常 ○ 总是
D.16 Were physicians explicitly encouraged to disclose medical errors to patients or their family members? (7)<br>○ Never ○ Rarely ○ Sometimes ○ Usually ○ Always	如果医疗过程中出现错误，工作团队会明确的鼓励医生们向患者或家属说明实际情况。<br>○ 从不 ○ 很少 ○ 有时 ○ 经常 ○ 总是

D.17	This workgroup engages in productive conflict and debate during discussions.<br>○ highly inaccurate ○ inaccurate ○ somewhat inaccurate ○ not sure ○ somewhat accurate ○ accurate ○ highly accurate	在讨论过程中，团队成员之间会有建设性的冲突与争论。<br>○ 很不准确 ○ 不准确 ○ 部分不准确 ○ 不确定 ○ 部分准确 ○ 准确 ○ 很准确
D.18	In this workgroup, we frequently identify and discuss underlying assumptions that might affect key decisions.<br>○ highly inaccurate ○ inaccurate ○ somewhat inaccurate ○ not sure ○ somewhat accurate ○ accurate ○ highly accurate	您所在的工作团队中，成员们常常会明确那些会影响关键决定的假定条件，并进行讨论。<br>○ 很不准确 ○ 不准确 ○ 部分不准确 ○ 不确定 ○ 部分准确 ○ 准确 ○ 很准确
D.19	The hospital has the resources and information it needs to reduce 30-day mortality rates in patients with AMI. (51)<br>○ Never ○ Rarely ○ Sometimes ○ Usually ○ Always	您所在的医院掌握了足够的资源和信息，可以用来降低 AMI 患者的死亡率。<br>○ 从不 ○ 很少 ○ 有时 ○ 经常 ○ 总是
D.20	Senior-level administration is supportive of efforts to improve AMI care. (52)<br>○ Never ○ Rarely ○ Sometimes ○ Usually ○ Always	您所在的医院的管理层支持改善 AMI 诊疗的工作。<br>○ 从不 ○ 很少 ○ 有时 ○ 经常 ○ 总是
D.21	There is simply no time for reflection in this workgroup.<br>○ highly inaccurate ○ inaccurate ○ somewhat inaccurate ○ not sure ○ somewhat accurate ○ accurate ○ highly accurate	您所在的团队中的成员没有时间来对工作进行反思。<br>○ 很不准确 ○ 不准确 ○ 部分不准确 ○ 不确定 ○ 部分准确 ○ 准确 ○ 很准确
D.22	In this workgroup, people are too busy to invest time in improvement.<br>○ highly inaccurate ○ inaccurate ○ somewhat inaccurate ○ not sure ○ somewhat accurate ○ accurate ○ highly accurate	您所在的团队中的成员工作太忙，所以没有时间对工作进行改进。<br>○ 很不准确 ○ 不准确 ○ 部分不准确 ○ 不确定 ○ 部分准确 ○ 准确 ○ 很准确
D.23	My manager(s) establish(es) forums for and provide(s) time and resources for identifying problems and organizational challenges.<br>○ highly inaccurate ○ inaccurate ○ somewhat inaccurate ○ not sure ○ somewhat accurate ○ accurate ○ highly accurate	您所在的医院的部门主管（们）会组织讨论会，并提供相应的时间和资源来，**明确临床工作中存在的问题和部门面临的挑战**。<br>○ 很不准确 ○ 不准确 ○ 部分不准确 ○ 不确定 ○ 部分准确 ○ 准确 ○ 很准确

D.24	My manager(s) establish(es) forums for and provide(s) time and resources for reflecting and improving on past performance.<br>○ highly inaccurate ○ inaccurate ○ somewhat inaccurate ○ not sure ○ somewhat accurate ○ accurate ○ highly accurate	您所在的医院的部门主管（们）会组织讨论会，并提供相应的时间和资源来，**反思过去的诊疗表现并进行改进**。<br>○ 很不准确 ○ 不准确 ○ 部分不准确 ○ 不确定 ○ 部分准确 ○ 准确 ○ 很准确
D.25	My manager(s) listen(s) attentively.<br>○ highly inaccurate ○ inaccurate ○ somewhat inaccurate ○ not sure ○ somewhat accurate ○ accurate ○ highly accurate	您所在的医院的部门主管（们）会很专心的听取别人的意见。<br>○ 很不准确 ○ 不准确 ○ 部分不准确 ○ 不确定 ○ 部分准确 ○ 准确 ○ 很准确
D.26	My manager(s) invite(s) input from others in discussions.<br>○ highly inaccurate ○ inaccurate ○ somewhat inaccurate ○ not sure ○ somewhat accurate ○ accurate ○ highly accurate	您所在的医院的部门主管（们）在讨论中会邀请其他人发表意见。<br>○ 很不准确 ○ 不准确 ○ 部分不准确 ○ 不确定 ○ 部分准确 ○ 准确 ○ 很准确
D.27	This workgroup experiments frequently with new product/service offerings.<br>○ highly inaccurate ○ inaccurate ○ somewhat inaccurate ○ not sure ○ somewhat accurate ○ accurate ○ highly accurate	您所在的工作团队经常会尝试其他单位提供的**与疾病诊断治疗相关的新的产品或服务**。<br>○ 很不准确 ○ 不准确 ○ 部分不准确 ○ 不确定 ○ 部分准确 ○ 准确 ○ 很准确
D.28	This workgroup experiments frequently with new ways of working.<br>○ highly inaccurate ○ inaccurate ○ somewhat inaccurate ○ not sure ○ somewhat accurate ○ accurate ○ highly accurate	您所在的工作团队经常会尝试**新的工作方法（包括工作和组织流程、诊断治疗措施和方法）**。<br>○ 很不准确 ○ 不准确 ○ 部分不准确 ○ 不确定 ○ 部分准确 ○ 准确 ○ 很准确
D.29	This workgroup frequently employs pilot projects or simulations when trying our new ideas.<br>○ highly inaccurate ○ inaccurate ○ somewhat inaccurate ○ not sure ○ somewhat accurate ○ accurate ○ highly accurate	您所在的工作团队常常会通过预实验或者采用场景模拟的方法对团队成员们的新想法进行尝试时（包括临床工作和组织流程、诊断治疗措施和方法）。<br>○ 很不准确 ○ 不准确 ○ 部分不准确 ○ 不确定 ○ 部分准确 ○ 准确 ○ 很准确

D.30	This workgroup has a formal process for conducting and evaluating experiments or new ideas.<br>○ highly inaccurate ○ inaccurate ○ somewhat inaccurate ○ not sure ○ somewhat accurate ○ accurate ○ highly accurate	您所在的工作团队在对新的工作方法（包括临床工作和组织流程、诊断治疗措施和方法）的进行试验和评价的过程中，有正规的实施方案和流程。<br>○ 很不准确 ○ 不准确 ○ 部分不准确 ○ 不确定 ○ 部分准确 ○ 准确 ○ 很准确
D.31	Experienced employees in this workgroup receive training when new initiatives are launched.<br>○ highly inaccurate ○ inaccurate ○ somewhat inaccurate ○ not sure ○ somewhat accurate ○ accurate ○ highly accurate	您所在的工作团队中，**当某项新措施开始实施时**，有经验的成员将会接受相关的培训。<br>○ 很不准确 ○ 不准确 ○ 部分不准确 ○ 不确定 ○ 部分准确 ○ 准确 ○ 很准确
D.32	Experienced employees in this workgroup receive training when shifting to a new position.<br>○ highly inaccurate ○ inaccurate ○ somewhat inaccurate ○ not sure ○ somewhat accurate ○ accurate ○ highly accurate	您所在的工作团队中，**当有经验的员工调任到新的岗位时**，将会接受相关的培训。<br>○ 很不准确 ○ 不准确 ○ 部分不准确 ○ 不确定 ○ 部分准确 ○ 准确 ○ 很准确
D.33	Newly hired employees in this workgroup receive adequate training.<br>○ highly inaccurate ○ inaccurate ○ somewhat inaccurate ○ not sure ○ somewhat accurate ○ accurate ○ highly accurate	您所在的工作团队中，新员工将会接受足够的培训。<br>○ 很不准确 ○ 不准确 ○ 部分不准确 ○ 不确定 ○ 部分准确 ○ 准确 ○ 很准确
D.34	Did your hospital provide training to EMS providers about AMI care? (17)<br>○ Yes, about monthly<br>○ Yes, about quarterly<br>○ Yes, about annually<br>○ Yes, other: __________<br>○ No<br>○ Unknown	您所在的医院会给急救中心的工作人员提供有关 AMI 诊疗的培训。<br>○ 是，大概每月都有<br>○ 是，大概每季度都有<br>○ 是，大概每年都有<br>○ 是，其他：______________________<br>○ 否<br>○ 不清楚

D.35	This workgroup has forums for meeting with and learning from: Experts from outside the organization.<br>○ highly inaccurate ○ inaccurate ○ somewhat inaccurate ○ not sure ○ somewhat accurate ○ accurate ○ highly accurate	您所在的工作团队会组织讨论会，邀请院外的专家来讲课。<br>○ 很不准确 ○ 不准确 ○ 部分不准确 ○ 不确定 ○ 部分准确 ○ 准确 ○ 很准确
D.36	This workgroup has forums for meeting with and learning from: Experts from other departments/teams/divisions.<br>○ highly inaccurate ○ inaccurate ○ somewhat inaccurate ○ not sure ○ somewhat accurate ○ accurate ○ highly accurate	您所在的工作团队会组织讨论会，邀请院内其他科室、团队或部门的专家来讲课。<br>○ 很不准确 ○ 不准确 ○ 部分不准确 ○ 不确定 ○ 部分准确 ○ 准确 ○ 很准确
D.37	This workgroup has forums for meeting with and learning from: Customers/clients.<br>○ highly inaccurate ○ inaccurate ○ somewhat inaccurate ○ not sure ○ somewhat accurate ○ accurate ○ highly accurate	您所在的工作团队会组织讨论会，邀请患者座谈讨论，并向他们学习。<br>○ 很不准确 ○ 不准确 ○ 部分不准确 ○ 不确定 ○ 部分准确 ○ 准确 ○ 很准确
D.38	This workgroup regularly conducts post-audits, after-action reviews, and debriefings.<br>○ highly inaccurate ○ inaccurate ○ somewhat inaccurate ○ not sure ○ somewhat accurate ○ accurate ○ highly accurate	您所在的工作团队会定期对工作进行事后考察、回顾和简报。<br>○ 很不准确 ○ 不准确 ○ 部分不准确 ○ 不确定 ○ 部分准确 ○ 准确 ○ 很准确
D.39	Did your hospital have regular 'morbidity and mortality' conferences (or another educational session) for discussing individual cases involving patients with AMI? (5)<br>○ Never ○ Rarely ○ Sometimes ○ Usually ○ Always	您所在的医院会定期组织有关的会议（或其他的教育课程）来讨论包括 AMI 在内的病例个案。<br>○ 从不 ○ 很少 ○ 有时 ○ 经常 ○ 总是

D.40	Did your hospital review the deaths of patients with AMI? (4a)<br>○ No, we did not review these cases **(go to D44)**<br>○ Yes, we reviewed only deaths with potential quality issues (i.e., unexpected deaths)<br>○ Yes, we reviewed *all* deaths<br>○ Other, please specify: ___________<br>○ Unknown	您所在的医院会回顾分析 AMI 患者的死亡个案吗？<br>○ 否，我们不评估这类死亡 **（转至 D44）**<br>○ 是，我们只评估与潜在的质量问题相关的死亡（比如，意外死亡）<br>○ 是，我们评估所有的死亡<br>○ 其他，请说明：____________<br>○ 不清楚
D.41	Did your hospital have a **designated person or group** to review the deaths of patients with AMI (i.e., on an individual case level) that occurred during hospitalization? (4)<br>○ Never ○ Rarely ○ Sometimes ○ Usually ○ Always	您所在的医院是否有专门指定的工作人员或团队回顾分析在住院期间发生的 AMI 患者的死亡个案？<br>○ 从不 ○ 很少 ○ 有时 ○ 经常 ○ 总是
D.42	How long after the occurrence of the death were the cases typically reviewed? (4b)<br>○ Within one week of the death<br>○ Within one month of the death<br>○ Within 3 months of the death<br>○ Other, please specify: ____________________________<br>○ We did not have a set timeframe for reviewing these cases<br>○ Unknown	您所在的医院通常会在这些死亡病例个案发生后多久进行回顾分析？<br>○ 死亡后的一周内<br>○ 死亡后的一个月内<br>○ 死亡后的三个月内<br>○ 其他，请说明：_______________<br>○ 我们对评估这些个案没有一个具体的时间要求<br>○ 不清楚

D.43	Who usually reviewed these cases? (4c)<br>a. Senior management of the hospital<br>○ Never ○ Rarely ○ Sometimes ○ Usually ○ Always<br>b. Cardiology chiefs<br>○ Never ○ Rarely ○ Sometimes ○ Usually ○ Always<br>c. Nursing directors<br>○ Never ○ Rarely ○ Sometimes ○ Usually ○ Always<br>d. Other physicians participating in the care of patients with AMI<br>○ Never ○ Rarely ○ Sometimes ○ Usually ○ Always<br>e. Quality Improvement/Quality Management department staff<br>○ Never ○ Rarely ○ Sometimes ○ Usually ○ Always	通常谁会回顾分析这些案例？<br>医院的管理层<br>○ 从不 ○ 很少 ○ 有时 ○ 经常 ○ 总是<br>a. 心脏科主任<br>○ 从不 ○ 很少 ○ 有时 ○ 经常 ○ 总是<br>b. 护理部主任<br>○ 从不 ○ 很少 ○ 有时 ○ 经常 ○ 总是<br>c. 其他参与到 AMI 患者诊疗过程的医生<br>○ 从不 ○ 很少 ○ 有时 ○ 经常 ○ 总是<br>d. 医疗质量改进/质量管理部门的员工<br>○ 从不 ○ 很少 ○ 有时 ○ 经常 ○ 总是
D.44	Did your hospital have a **designated person or group** to review any of the following **adverse events** in patients with AMI (i.e., on an individual case level)? (6)<br>a. Sentinel events (unexpected occurrence involving death or serious physical or psychological injury) that occurred during hospitalization<br>○ Never ○ Rarely ○ Sometimes ○ Usually ○ Always<br>b. Unexpected transfers from a floor (non-monitored unit) to an intensive are unit<br>○ Never ○ Rarely ○ Sometimes ○ Usually ○ Always<br>c. Catastrophic complications that occurred immediately after discharge from the hospital<br>○ Never ○ Rarely ○ Sometimes ○ Usually ○ Always	您所在的医院是否有专门指定的工作人员或团队回顾分析 AMI 患者中的以下不良事件个案吗？<br>a. 发生在住院期间的预期外不良事件（涉及到死亡或严重身体/心理创伤）<br>○ 从不 ○ 很少 ○ 有时 ○ 经常 ○ 总是<br>b. 预期外的从普通住院病房（非监护病房）转移到重症监护室<br>○ 从不 ○ 很少 ○ 有时 ○ 经常 ○ 总是<br>c. 出院后即刻发生的严重性并发症<br>○ 从不 ○ 很少 ○ 有时 ○ 经常 ○ 总是

D.45 How long after the occurrence of these adverse events were the cases typically reviewed? (6a)<br>o Within one week of the adverse event<br>o Within one month of the adverse event<br>o Within 3 months of the adverse event<br>o Other, please specify: ____________<br>o We did not have a set timeframe for reviewing these cases<br>o Unknown	您所在的医院通常会在这些不良事件病例个案发生后多久进行回顾分析？<br>o 事件后的一周内<br>o 事件后的一个月内<br>o 事件后的三个月内<br>o 其他，请说明：_________________<br>o没有具体的时间要求<br>o 不清楚
D.46 Who usually reviewed these cases? (6b)<br>a. Senior management of the hospital<br>o Never o Rarely o Sometimes o Usually o Always<br>b. Cardiology chiefs<br>o Never o Rarely o Sometimes o Usually o Always<br>c. Nursing directors<br>o Never o Rarely o Sometimes o Usually o Always<br>d. Other physicians participating in the care of patients with AMI<br>o Never o Rarely o Sometimes o Usually o Always<br>e. Quality Improvement/Quality Management department staff<br>o Never o Rarely o Sometimes o Usually o Always<br>f. Other, please specify: ___	通常谁会回顾分析这些案例？<br>医院的管理层<br>o 从不 o 很少 o 有时 o 经常 o 总是<br>a. 心脏科主任<br>o 从不 o 很少 o 有时 o 经常 o 总是<br>b. 护理部主任<br>o 从不 o 很少 o 有时 o 经常 o 总是<br>c. 其他参与到 AMI 患者诊疗过程的医生<br>o 从不 o 很少 o 有时 o 经常 o 总是<br>d. 医疗质量改进/质量管理部门的员工<br>o 从不 o 很少 o 有时 o 经常 o 总是<br>e. 其他，请说明：___

D.47	Did your hospital use root cause analysis or a similar method to understand the following problems in AMI care?<br>a. Poor adherence to the core medication (i.e., anti-platelet agents) measures<br>○ Never ○ Rarely ○ Sometimes ○ Usually ○ Always<br>b. Delay to fibrinolytic therapy or percutaneous coronary intervention (PCI)<br>○ Never ○ Rarely ○ Sometimes ○ Usually ○ Always	您所在的医院会使用根本原因分析法或其他类似的方法来了解 AMI 诊疗中的下列问题吗？<br>a. 未能按照指南要求使用关键药物（如抗血小板药）治疗<br>○ 从不 ○ 很少 ○ 有时 ○ 经常 ○ 总是<br>b. 从就诊到实施溶栓治疗或经皮冠状动脉介入治疗（PCI）的时间延误<br>○ 从不 ○ 很少 ○ 有时 ○ 经常 ○ 总是
D.48	Did your hospital review data on **30-day mortality rates** (deaths occurring within 30 days of admission, including both inpatient and post-discharge deaths) in patients admitted with AMI (Check all that apply) (10)<br>□ Yes, through the medical insurance data system<br>□ Yes, through a regional database system<br>□ Yes, we internally collect our own data on deaths<br>□ Yes, other, please specify: ___<br>□ No **[Skip to D52]**<br>□ Unknown	您所在的医院会回顾评估 AMI 住院治疗的患者院内死亡率吗？***[可多选]***<br>□ 是，通过医保中心获得数据<br>□ 是，通过一个地区性数据系统<br>□ 是，我们会内部收集有关死亡的数据<br>□ 是，其他，请说明：___<br>□ 否 **[转至 D52]**<br>□ 不清楚

D.49	How quickly were **mortality rates** in patients with AMI available to your hospital (i.e., what was the most current data available to your hospital)? (10a)<br>○ Within 6 months of care delivery<br>○ 6 months to 1 year after care delivery<br>○ 1 - 2 years after care delivery<br>○ Less frequently than 2 years of care delivery<br>○ Unknown	您所在的医院最快能在什么时候获得 AMI 患者死亡率的相关数据（也就是说，您所在的医院最近的 AMI 患者死亡率数据是什么时候的）？<br>○ 提供医疗服务后 6 个月内<br>○ 提供医疗服务后 6 个月到 1 年<br>○ 提供医疗服务后 1-2 年<br>○ 提供医疗服务后 2 年以上<br>○ 不清楚
D.50	Did your hospital regularly compare its performance to other hospitals on either **inpatient** in patients with AMI? (14)<br>○ Never ○ Rarely ○ Sometimes ○ Usually ○ Always	您所在的医院会定期的将 AMI 患者的住院死亡率与其他医院进行比较吗？<br>○ 从不 ○ 很少 ○ 有时 ○ 经常 ○ 总是
D.51	Did your hospital have **efforts** to improve any of the following inpatient acute myocardial infarction (AMI) quality measures? (1)<br>a. Adherence to the core medication (i.e., anti-platelet agents) measures<br>○ Never ○ Rarely ○ Sometimes ○ Usually ○ Always<br>b. Time to fibrinolytic therapy or percutaneous coronary intervention (PCI)<br>○ Never ○ Rarely ○ Sometimes ○ Usually ○ Always	您所在的医院是否曾采取措施努力去改善下列 AMI 患者住院治疗的质量指标？<br>a. 按照指南要求使用关键药物（如抗血小板药）治疗的比例<br>○ 从不 ○ 很少 ○ 有时 ○ 经常 ○ 总是<br>b. 从就诊到实施溶栓治疗或经皮冠状动脉介入治疗（PCI）的时间<br>○ 从不 ○ 很少 ○ 有时 ○ 经常 ○ 总是

D.52	Beyond these quality measures, did your hospital initiate **efforts** to improve any of the following in patients admitted with AMI? (2)<br>a. Inpatient mortality in patients with AMI<br>○ Never ○ Rarely ○ Sometimes ○ Usually ○ Always<br>b. **Post-discharge** mortality (death occurring after discharge, but within 30 days of admission) in patients with AMI<br>○ Never ○ Rarely ○ Sometimes ○ Usually ○ Always<br>c. Readmission within 30 days from prior admission in patients with AMI<br>○ Never ○ Rarely ○ Sometimes ○ Usually ○ Always	除了这些质量指标外，您所在的医院是否采取措施改善以下 AMI 患者住院治疗的指标。<br>a. AMI 患者的住院死亡率<br>○ 从不 ○ 很少 ○ 有时 ○ 经常 ○ 总是<br>b. AMI 患者的出院后死亡率（出院后，但仍在入院后的 30 天内）<br>○ 从不 ○ 很少 ○ 有时 ○ 经常 ○ 总是<br>c. AMI 患者在上一次入院后的 30 天内再次住院率<br>○ 从不 ○ 很少 ○ 有时 ○ 经常 ○ 总是
D.53	Did your hospital have a quality improvement **team(s)** devoted to improving: (3)<br>a. Inpatient mortality in patients with AMI<br>○ Never ○ Rarely ○ Sometimes ○ Usually ○ Always<br>b. Post-discharge mortality (death occurring after discharge, but within 30 days of admission) in patients with AMI<br>○ Never ○ Rarely ○ Sometimes ○ Usually ○ Always	您所在的医院有一个或多个质量改善团队，以致力于改善下列方面吗？<br>a. AMI 患者的住院死亡率<br>○ 从不 ○ 很少 ○ 有时 ○ 经常 ○ 总是<br>b. AMI 患者的出院后死亡率（出院后，但仍在入院后的 30 天内）<br>○ 从不 ○ 很少 ○ 有时 ○ 经常 ○ 总是

D.54	3a. Please indicate members of either the inpatient or post-discharge mortality **team(s)**.<br>a. Senior management of the hospital<br>○ Never ○ Rarely ○ Sometimes ○ Usually ○ Always<br>b. Hospital governing board<br>○ Never ○ Rarely ○ Sometimes ○ Usually ○ Always<br>c. Chief of cardiology<br>○ Never ○ Rarely ○ Sometimes ○ Usually ○ Always<br>d. Nursing directors<br>○ Never ○ Rarely ○ Sometimes ○ Usually ○ Always<br>e. Other physicians participating in the care of patients with AMI<br>○ Never ○ Rarely ○ Sometimes ○ Usually ○ Always<br>f. Quality Improvement/Quality Management department staff<br>○ Never ○ Rarely ○ Sometimes ○ Usually ○ Always<br>g. Other please specify: _________________________	请指出在住院死亡率或出院后死亡率团队里的人员构成。<br>a. 医院的管理层<br>○ 从不 ○ 很少 ○ 有时 ○ 经常 ○ 总是<br>b. 医院董事会<br>○ 从不 ○ 很少 ○ 有时 ○ 经常 ○ 总是<br>c. 心脏科主任<br>○ 从不 ○ 很少 ○ 有时 ○ 经常 ○ 总是<br>d. 护理部主任<br>○ 从不 ○ 很少 ○ 有时 ○ 经常 ○ 总是<br>e. 其他参与到 AMI 患者护理的医生<br>○ 从不 ○ 很少 ○ 有时 ○ 经常 ○ 总是<br>f. 质量改善/质量管理部门员工<br>○ 从不 ○ 很少 ○ 有时 ○ 经常 ○ 总是<br>g. 其他，请说明：____
D.55	Nurses are engaged in efforts to improve AMI care. (53)<br>○ Never ○ Rarely ○ Sometimes ○ Usually ○ Always	在您所在的工作团队中，护士会参与为改善 AMI 诊疗而开展的工作。<br>○ 从不 ○ 很少 ○ 有时 ○ 经常 ○ 总是
D.56	Cardiologists are engaged in efforts to improve AMI care. (54)<br>○ Never ○ Rarely ○ Sometimes ○ Usually ○ Always	心脏科医生会参与为改善 AMI 诊疗而开展的工作。<br>○ 从不 ○ 很少 ○ 有时 ○ 经常 ○ 总是
D.57	Emergency medicine physicians are engaged in efforts to improve AMI care. (55)<br>○ Never ○ Rarely ○ Sometimes ○ Usually ○ Always	急诊科医生会参与为改善 AMI 诊疗而开展的工作。<br>○ 从不 ○ 很少 ○ 有时 ○ 经常 ○ 总是

D.58	Did your hospital have one or more physician champions focused on improving either **inpatient** or **30-day mortality** in patients with AMI? (12)<br>o Never o Rarely o Sometimes o Usually o Always	您所在的医院是否有一个或多个医生作为负责人致力于改善 AMI 患者的住院死亡率或 30 天死亡率？<br>o 从不 o 很少 o 有时 o 经常 o 总是
D.59	Did your hospital have one or more nurse champions focused on improving either **inpatient** or **30-day mortality** in patients with AMI? (13)<br>o Never o Rarely o Sometimes o Usually o Always	您所在的医院是否有一个或多个护士作为负责人致力于改善 AMI 患者的住院死亡率或 30 天死亡率？<br>o 从不 o 很少 o 有时 o 经常 o 总是
D.60	After we make changes to improve AMI care, we fail to evaluate their effectiveness. (67)<br>o Never o Rarely o Sometimes o Usually o Always	您所在的工作团队做了一些工作来改善 AMI 患者治疗质量，但却无法评估这些措施的效果。<br>o 从不 o 很少 o 有时 o 经常 o 总是
D.61	Did cardiology and emergency department staff meet together to review care for patients with AMI? (15)<br>o Yes, about monthly<br>o Yes, about quarterly<br>o Yes, about annually<br>o Yes, other: ____________________<br>o No **[Skip to D63]**<br>o Unknown	心脏科和急诊科的工作人员会在一起回顾评估 AMI 患者的诊疗吗？<br>o 是，大概每月都有<br>o 是，大概每季度都有<br>o 是，大概每年都有<br>o 是，其他____________________<br>o 否 **[转至 D63]**<br>o 不清楚

D.62 What was typically discussed at these meetings? (15a).<br>a. Care of patients with ST-elevation myocardial infarction (STEMI)<br>○ Never ○ Rarely ○ Sometimes ○ Usually ○ Always<br>b. Care of patients with Unstable Angina/**non**-STEMI (NSTEMI)<br>○ Never ○ Rarely ○ Sometimes ○ Usually ○ Always<br>c. Care of patients with chest pain, in general<br>○ Never ○ Rarely ○ Sometimes ○ Usually ○ Always	在这些回顾评估过程中，一般会讨论哪些问题？<br>a. ST 段抬高型心肌梗死（STEMI）患者的诊疗<br>○ 从不 ○ 很少 ○ 有时 ○ 经常 ○ 总是<br>b. 不稳定性心绞痛/非 ST 段抬高型心肌梗死（NSTEMI）患者的诊疗<br>○ 从不 ○ 很少 ○ 有时 ○ 经常 ○ 总是<br>c. 一般胸痛症状的患者的诊疗<br>○ 从不 ○ 很少 ○ 有时 ○ 经常 ○ 总是
D.63 Did clinicians from your hospital meet with emergency medical system (EMS) providers to review the care of patients with AMI? (16)<br>○ Yes, about monthly<br>○ Yes, about quarterly<br>○ Yes, about annually<br>○ Yes, other: _____________________<br>○ No<br>○ Unknown	您所在医院的医生会与急救中心的工作人员在一起回顾评估对 AMI 患者的诊疗吗？<br>○ 是，大概每月都有<br>○ 是，大概每季度都有<br>○ 是，大概每年都有<br>○ 是，其他：_____________________<br>○ 否<br>○ 不清楚
D.64 There is good coordination among the different departments involved with the care of patients with AMI. (62)<br>○ Never ○ Rarely ○ Sometimes ○ Usually ○ Always	医院内参与 AMI 患者治疗的不同部门间能够有效协作。<br>○ 从不 ○ 很少 ○ 有时 ○ 经常 ○ 总是
D.65 Departments caring for patients with AMI (e.g., cardiology, emergency medicine) communicate easily with each other.(64)<br>○ Never ○ Rarely ○ Sometimes ○ Usually ○ Always	医院内参与 AMI 患者治疗的不同部门（如心脏科，急诊科）间相互沟通很顺畅。<br>○ 从不 ○ 很少 ○ 有时 ○ 经常 ○ 总是
D.66 Clinicians caring for patients with AMI share new evidence-based approaches with the AMI team.(63)<br>○ Never ○ Rarely ○ Sometimes ○ Usually ○ Always	参与 AMI 患者治疗的医生会与整个 AMI 治疗团队分享新的循证医学方法。<br>○ 从不 ○ 很少 ○ 有时 ○ 经常 ○ 总是

D.67	Which best describes the quality of your interaction with hospitals **that referred patients to you with AMI**?(18)<br>○ Very collaborative (we shared data along with strategies for improving AMI care) ○ Somewhat collaborative (we communicated regularly, but we did not share data and strategies)<br>○ Not collaborative (we had no or minimal contact with the referring hospital/s)<br>○ Not applicable **[Skip to D69]**	关于您和将 AMI 患者转诊给您的医院之间的交流互动的情况如何？<br>○ 非常合作（我们互享数据以及改善 AMI 诊疗的策略）<br>○ 部分合作（我们定期交流，但我们不分享数据和策略）<br>○ 不合作（我们与涉及到的医院没有或有最少的接触）<br>○ 不适用（没有医院向您所在的医院转诊 AMI 患者） **[转至 D69]**
D.68	Did your hospital routinely give feedback to the referring hospital/s on any of the following? (18a.)<br>a. Time to transfer<br>○ Never ○ Rarely ○ Sometimes ○ Usually ○ Always<br>b. AMI-related procedures performed<br>○ Never ○ Rarely ○ Sometimes ○ Usually ○ Always<br>c. Patient outcome<br>○ Never ○ Rarely ○ Sometimes ○ Usually ○ Always<br>d. Other please specify: ___________________________	您所在的医院会常规的将有关下列各项的反馈给将患者转诊来的医院吗？<br>a. 转移患者花费的时间<br>○ 从不 ○ 很少 ○ 有时 ○ 经常 ○ 总是<br>b. 实施的 AMI 相关治疗<br>○ 从不 ○ 很少 ○ 有时 ○ 经常 ○ 总是<br>c. 患者的治疗结局<br>○ 从不 ○ 很少 ○ 有时 ○ 经常 ○ 总是<br>d. 其他，请说明：______________________
D.69	Which best describes the quality of your interaction with hospitals **that you referred patients to with AMI**? (19)<br>○ Very collaborative (we shared data along with strategies for improving AMI care) ○ Somewhat collaborative (we communicated regularly, but we did not share data and strategies)<br>○ Not collaborative (we had no or minimal contact with hospitals in our region)<br>○ Not applicable	关于您和您将 AMI 患者转诊去的医院之间的交流互动情况如何？<br>○ 非常合作（我们互享数据以及改善 AMI 诊疗的策略）<br>○ 部分合作（我们定期交流，但我们不分享数据和策略）<br>○ 不合作（我们与区域内的医院没有或有很少的接触）<br>○ 不适用（您所在的医院不向其他医院转诊 AMI 患者）

D.70 Was your hospital part of a regional effort or consortium of hospitals to improve AMI care? (20)<br>○ Never ○ Rarely ○ Sometimes ○ Usually ○ Always	您所在的医院参与了为改善 AMI 诊疗而组织的地区性医院合作项目？<br>○ 从不 ○ 很少 ○ 有时 ○ 经常 ○ 总是

Thank you for your time and participation.

非常感谢您的参与和宝贵的时间！
